# Ras/ERK-signalling promotes tRNA synthesis and growth via the RNA polymerase III repressor Maf1 in *Drosophila*

**DOI:** 10.1371/journal.pgen.1007202

**Published:** 2018-02-05

**Authors:** Shrivani Sriskanthadevan-Pirahas, Rujuta Deshpande, Byoungchun Lee, Savraj S. Grewal

**Affiliations:** Clark H Smith Brain Tumour Centre, Arnie Charbonneau Cancer Institute, Alberta Children’s Hospital Research Institute, and Department of Biochemistry and Molecular Biology Calgary, University of Calgary, Calgary, Alberta, Canada; University of Cambridge, UNITED KINGDOM

## Abstract

The small G-protein Ras is a conserved regulator of cell and tissue growth. These effects of Ras are mediated largely through activation of a canonical RAF-MEK-ERK kinase cascade. An important challenge is to identify how this Ras/ERK pathway alters cellular metabolism to drive growth. Here we report on stimulation of RNA polymerase III (Pol III)-mediated tRNA synthesis as a growth effector of Ras/ERK signalling in *Drosophila*. We find that activation of Ras/ERK signalling promotes tRNA synthesis both in vivo and in cultured *Drosophila* S2 cells. We also show that Pol III function is required for Ras/ERK signalling to drive proliferation in both epithelial and stem cells in *Drosophila* tissues. We find that the transcription factor Myc is required but not sufficient for Ras-mediated stimulation of tRNA synthesis. Instead we show that Ras signalling promotes Pol III function and tRNA synthesis by phosphorylating, and inhibiting the nuclear localization and function of the Pol III repressor Maf1. We propose that inhibition of Maf1 and stimulation of tRNA synthesis is one way by which Ras signalling enhances protein synthesis to promote cell and tissue growth.

## Author summary

The Ras oncogene is one of the primary drivers of cell and tissue growth in both normal development and in diseases such as cancer. In this report, we identify the stimulation of tRNA synthesis as an important mechanism by which Ras functions. Using fruit fly genetics, we show that Ras promotes tRNA synthesis by inhibiting Maf1, a protein that normally inhibits RNA polymerase III, the enzyme complex that stimulates tRNA synthesis. We further show that stimulation of tRNA synthesis is required for Ras to promote growth in two important cell types—stem cells and epithelial cells. This work provides new insight into mechanisms that are important for growth and that may contribute to cancer.

## Introduction

The Ras small G-protein is one of the key conserved regulators of cell growth and proliferation. Over three decades of research have defined the textbook model of how Ras is activated by growth factors to stimulate a core RAF kinase, MEK (Mitogen-activated protein kinase kinase) and ERK (Extracellular signal–regulated kinase) signalling cascade. Work in model organisms such as *Drosophila*, *C elegans* and mouse has shown how this Ras/ERK pathway coordinates tissue growth and patterning to control organ size during development and homeostatic growth in adults.

Given its central role in development it is not surprising that defects in Ras signalling contribute to disease. Most notably, activating mutations in Ras and RAF occur in a large percentage of cancers, and lead to hyper-activation of ERK, which drives tumour formation in both epithelial and stem cells [1]. Ras pathway mutations are also seen in several genetic developmental disorders–described collectively as RASopathies–often characterized by abnormal growth[2]. Understanding how Ras promotes cell proliferation and tissue growth is therefore an important concern in biology.

*Drosophila* has been a powerful model system to understand the biological roles of Ras signalling. In flies, Ras functions downstream of epidermal growth factor (EGF) and activation of its tyrosine kinase receptor (the EGFR). A series of genetic studies initiated over 25 years ago were pivotal in defining the canonical EGFR/Ras/ERK pathway in *Drosophila* (for reviews of this early work see:[3,4]). Extensive studies since then have established when, where and how the pathway is activated during the fly life cycle to control development. This work has emphasized the importance of Ras signalling in the control of cell growth and proliferation (e.g. [5–9]. Notably, during larval development Ras/ERK promotes EGFR-mediated cell proliferation and tissue growth in epithelial organs such as the imaginal discs, which eventually give rise to adult structures such as the legs, wings and eyes [10–14]. In addition, in the adult the EGFR/Ras/ERK signalling controls proliferation of stem cell populations to maintain homeostasis and promote regenerative growth [15–19].

How does Ras mediate these effects on cell and tissue growth? Most work on this area has focused on transcriptional effects of Ras signalling. Work in *Drosophila* has identified several transcription factors that are targeted by ERK such as *fos*, *capicua*, and *pointed*, and that regulate growth [19–22]. Ras signalling has also been shown to crosstalk with other transcriptional regulators of growth such as the *hippo/yorkie* pathway and *dMyc* [10,23–27]. These transcriptional effects control expression of metabolic and cell cycle genes important for growth[20,21]. Less is known, however, about how Ras/ERK may regulate mRNA translation to drive growth. The prevailing view, arising mostly from mammalian tissue culture experiments, is that ERK controls protein synthesis by stimulating the activity of translation initiation factors[28]. In particular, these effects are mediated via two ERK effector families—the MNK (MAP kinase-interacting serine/threonine-protein kinase) and RSK *(*ribosomal s6 kinase) kinases [28–30]. These kinases are important for cellular transformation and tumour growth in mammalian cells [31–34]. However, MNK and RSK mutants in mice and *Drosophila* have little growth or developmental phenotypes, and mouse MNK mutant cells show no alterations in protein synthesis [33–37]. These findings suggest Ras uses additional mechanisms to control translation and growth in vivo during animal development.

In this paper, we report that the Ras/ERK pathway can stimulate RNA polymerase III-dependent tRNA synthesis. We find that these effects are required for Ras to drive proliferation in both epithelial and stem cells. Finally, we show that ERK promotes tRNA synthesis by inhibiting the Pol III repressor Maf1. These findings suggest that stimulation of tRNA synthesis may be one way that Ras promotes mRNA translation to drive cell and tissue growth.

## Results

### Activation of Ras/ERK signalling leads to increased protein synthesis

**Fig 1:**
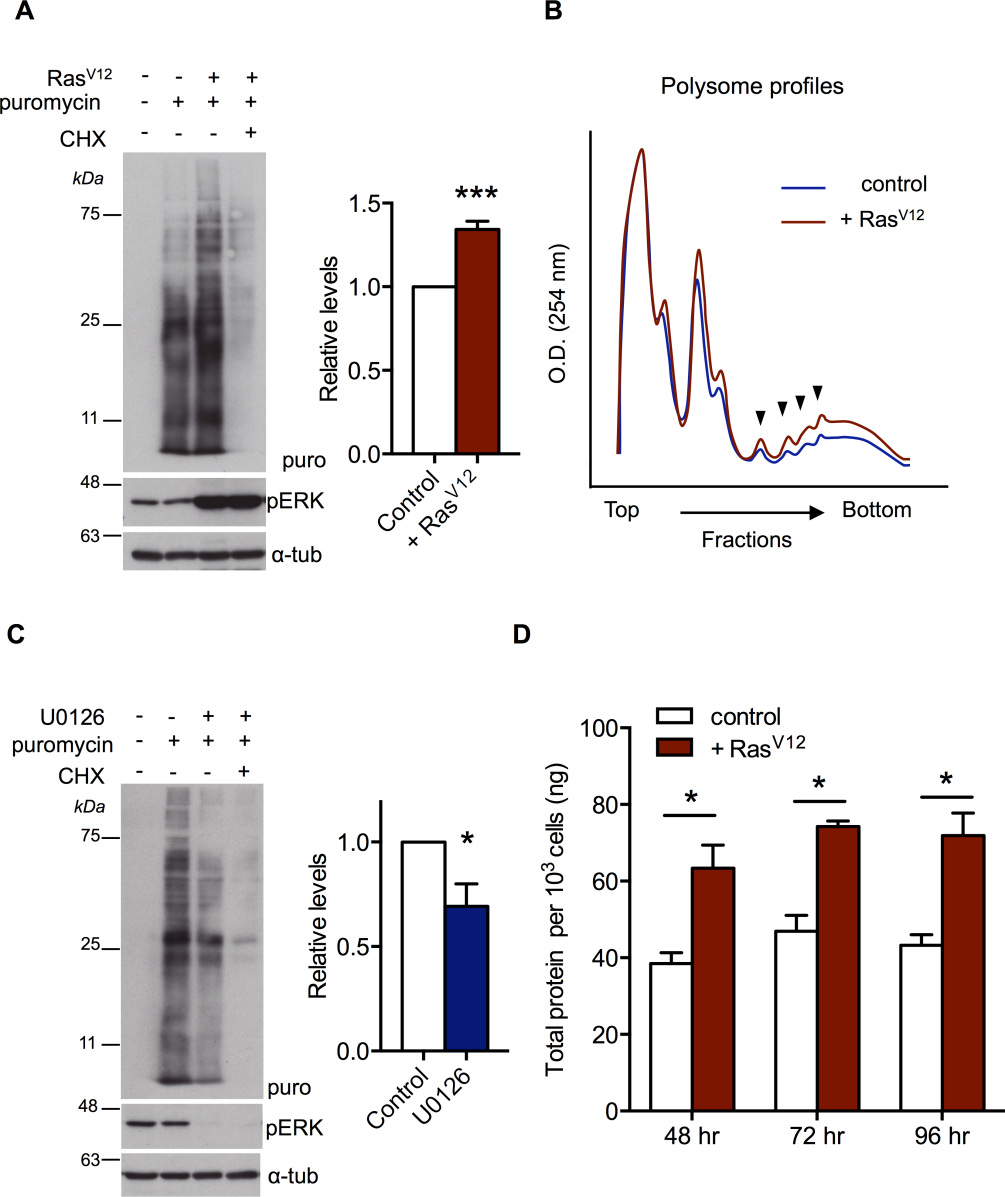
Activated Ras/ERK signalling pathway stimulates protein synthesis. (A) Left: Ras^V12^ expression was induced in cultured *Drosophila* S2 cells for 24 hrs. Cells were then incubated in puromycin for 30 min. Protein extracts were separated by SDS-PAGE and analyzed by western blot with an antibody to puromycin to measure the levels of puromycin-labelled peptides. Cycloheximide treatment was for 15 mins prior to addition of puromycin. A phospho-ERK immunoblot is shown as an indication of Ras/ERK signalling pathway activation. An alpha-tubulin immunoblot is shown as a loading control. Right: Experiments were performed in at least three biological replicates and western blots were quantified using NIH Image J software. Data are represented as relative levels (mean +/- SEM) compared to control (B) A Representative polysome profiles from control S2 cells (blue) and S2 cells with induced Ras^V12^ expression (red). Polysome peaks (arrowheads) in Ras^V12^ expressing cells were higher compared to controls, suggesting translation was increased. (C) Left: *Drosophila* S2 cells were treated with in the presence or absence of 10 μM U0126 for 2 hours at 25°C. Cells were then incubated in puromycin for 30 min. Protein extracts were separated by SDS-PAGE and analyzed by western blot with an antibody to puromycin to measure the levels of puromycin-labelled peptides. A phospho-ERK immunoblot is shown as an indication of Ras/ERK signalling pathway activation. An alpha-tubulin immunoblot is shown as a loading control. Right: Experiments were performed in at least three biological replicates and western blots were quantified using NIH Image J software. Data are represented as relative levels (mean +/- SEM) compared to control. (D) Ras^V12^ expression was induced in cultured *Drosophila* S2 cells for 48–96 hrs. Total protein content per 10^3^ cells was calculated using a Bradford assay. Data represent mean +/- SEM for at least three independent replicates per time point.

We first examined whether Ras signalling regulates protein synthesis in *Drosophila* S2 cells using a puromycin-labelling assay [38]. When a constitutively active Ras mutant (Ras^V12^) was expressed in *Drosophila* S2 cells using an inducible expression vector, we found an increase in protein synthesis, which was blocked by treatment of cells with cycloheximide (CHX), an inhibitor of mRNA translation (Fig 1A). Also, using polysome profiling to measure mRNA translation, we saw an increase in polysome levels in Ras^V12^ overexpressing cells when compared with control cells (Fig 1B). Conversely when we blocked Ras/ERK signalling by treating cells with the MEK inhibitor, U0126, protein synthesis was decreased (Fig 1C). Finally, we found that total protein content/cell increased after Ras^V12^ was overexpressed in S2 cells (Fig 1D). Our findings suggest that one way that the Ras/ERK signalling pathway may drive growth in *Drosophila* is by promoting protein synthesis.

### Ras/ERK signalling promotes tRNA synthesis

**Fig 2:**
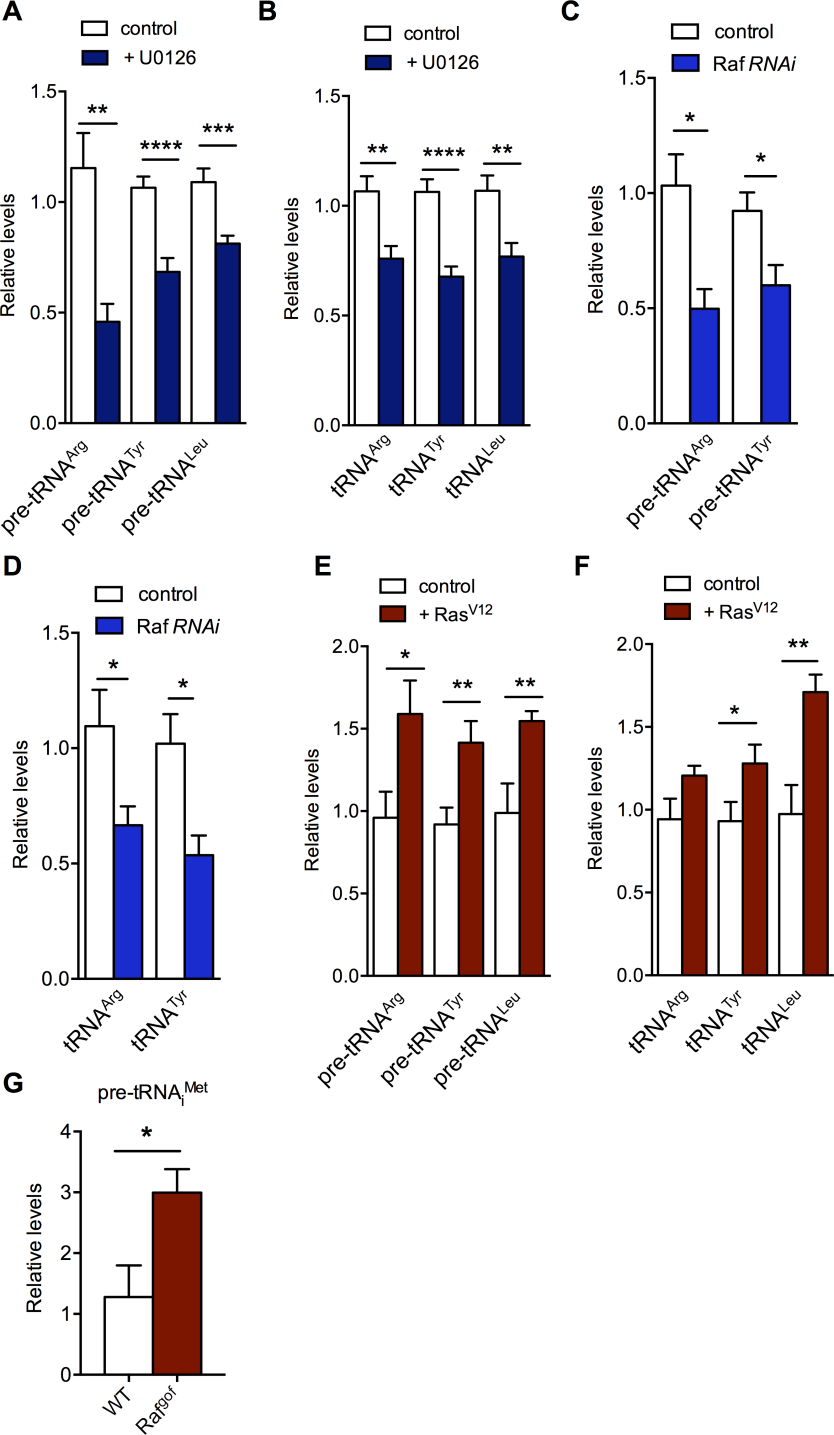
The Ras/ERK signalling pathway stimulates tRNA synthesis. (A, B) *Drosophila* S2 cells were treated with 10 μM U0126 for 2 hours. Total RNA was isolated and levels of either pre-tRNAs (A), or total tRNAs (B) measured by qRT-PCR. N = 15 independent samples per condition. (C, D) Raf was knocked down in *Drosophila* S2 cells by incubating cells with dsRNAs against *Raf*. Control cells were treated with dsRNA to GFP. Total RNA was isolated and levels of either pre-tRNAs (C), or total tRNAs (D) measured by qRT-PCR. N = 4 independent samples per condition. (E, F) Ras^V12^ expression was induced in *Drosophila* S2 cells for 24 hours. Total RNA was isolated and levels of either pre-tRNAs (E), or total tRNAs (F) measured by qRT-PCR. N = 9 independent samples per condition. (G) *UAS-Raf*^*gof*^ was expressed in imaginal tissues using the *esg-GAL4*^*ts*^ system. Control flies were *esg-GAL4*^*ts*^ flies crossed to *w*^*1118*^. Transgenes were induced by shifting larvae to 29°C at 48hrs of larval development, and then discs were dissected from wandering L3 stage larvae. Total RNA was isolated and levels of pre-tRNAs measured by qRT-PCR. N = 4 independent samples per condition. Data are presented as mean +/- SEM.

We previously identified the regulation of RNA Polymerase III and tRNA synthesis as a mechanism for controlling protein synthesis in *Drosophila* larvae [39,40]. We showed that these processes were regulated by TORC1 kinase signalling, and that they were important for driving tissue and body growth[39,40]. We were therefore interested in examining Ras signalling could also promote tRNA synthesis. We first used qRT-PCR to examine both pre-tRNA and total tRNA levels in S2 cells. We began by using the pharmacological MEK inhibitor U0126 to examine the effects of blocking Ras signalling. We found that treatment of S2 cells with UO126 lead to a decrease in levels of both pre-tRNAs and total tRNAs (Fig 2A and 2B). Also, using Northern blots, we saw that treatment with UO126 lead to reduced pre-tRNA and tRNA levels in S2 cells (S1A Fig). We also examined the effects of Ras pathway inhibition by using RNAi to knockdown Raf. We found that treatment of cells with dsRNA to Raf lead to reduced levels of both pre-tRNA and total tRNAs (Fig 2C and 2D). In contrast to Ras pathway inhibition, we found that Ras^V12^ (constitutively active Ras) overexpression lead to an increase in both pre-tRNA and mature tRNA levels as measured by both qRT-PCR (Fig 2E and 2F) and Northern blot (S1B Fig), indicating enhanced tRNA synthesis. In contrast, to these effects on tRNA levels, we found no effect of inhibiting Ras signalling on expression levels of TBP (which was previously reported[41]) or on levels of Brf1 or Trf1 –both of which are components of TFIIIB complex, which is required for Pol III recruitment to tRNA genes (S1C Fig). We also found that altering Ras signalling (either by MEK inhibition or overexpression of Ras^V12^) had no effect on levels of 5S or 7SL RNA in S2 cells (S1C and S1D Fig). Both of these genes transcribed by RNA polymerase III, but they are different Pol III gene types (type I and III respectively) that use a different set of core promoter factors compared to tRNA genes (which are type II Pol III genes). These finding suggest that the changes in tRNA synthesis we observed upon altering Ras signalling are not due to alterations in the levels of the basal transcriptional machinery required for tRNA transcription. These data also suggest Ras signalling may predominantly affect type II RNA pol III genes.

We also examined the effects of Ras signalling on tRNA levels in the developing wing imaginal discs. We used the temperature-sensitive *escargot-Gal4* (*esg-Gal4*^*ts*^) system, which allows for inducible transgenes expression in all imaginal tissues. When we overexpressed UAS-*Raf*^*gof*^ using this system, we found a marked increase in pre-tRNA levels in wing discs as measured by qRT-PCR on dissected wing discs (Fig 2G).

### Brf1 is required for Ras-induced tRNA synthesis and cell proliferation in wing discs

**Fig 3:**
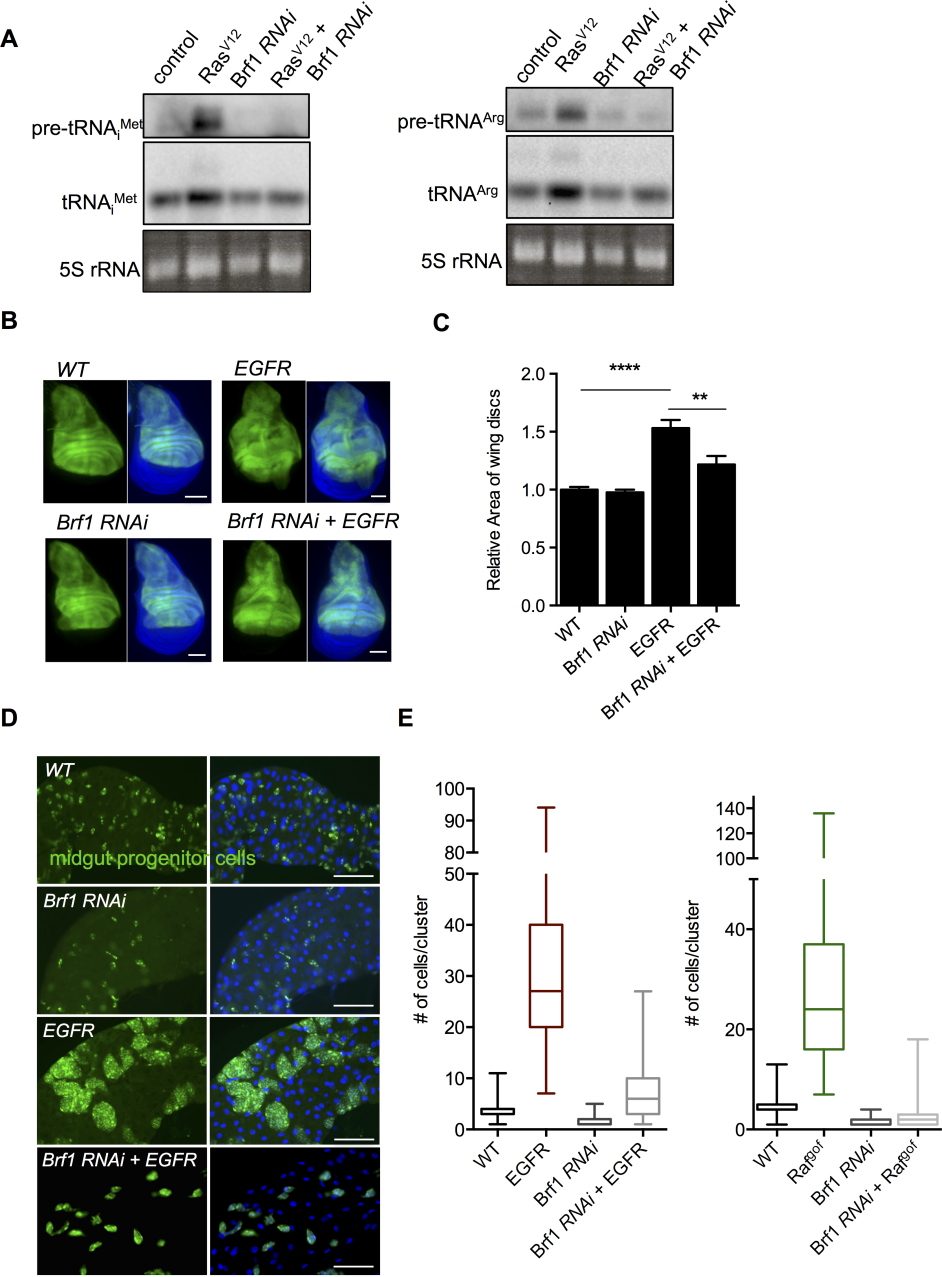
Brf1 is required for Ras-induced tRNA synthesis and growth in both wing imaginal discs and adult midgut progenitor cells (AMPs). (A). Ras^V12^ expression was induced in *Drosophila* S2 cells for 24 hours in either control cells or Brf1 knockdown cells, Brf1 was knocked down by incubating cells with dsRNA against Brf1. Control cells were treated with dsRNA to GFP. Total RNA was isolated with Trizol and analyzed by northern blotting using DIG-labelled antisense probes to tRNA^iMet^ or tRNA^Arg^. Ethidium bromide stained 5S rRNA band was used as a loading control. (B, C) *UAS-EGFR* and *UAS-Brf1 RNAi* were expressed, either alone or together, in the dorsal compartment of larval wing imaginal discs using an *ap-Gal4* driver. Control discs were from *ap-Gal4* crossed to *w*^*1118*^. Wing discs were dissected at the wandering L3 larval stage and the area of the GFP-marked dorsal compartment quantified using NIH imaging software (n > 50 wings per genotype, data presented as mean +/- SEM). Representative images are shown in (B), quantification of tissue area is shown in (C). (D) *UAS-EGFR* and *UAS-Brf1 RNAi* were expressed, either alone or together, in the *Drosophila* larval AMPs using the *esg-Gal4*^*ts*^ system. Larvae were shifted to 29°C at 24 hrs of development to induce transgene expression and dissected as L3 larvae. AMPs are marked *by UAS-GFP* expression. DNA is stained with Hoechst dye (blue). (E) The number of cells in each AMP cluster was quantified for each of the genotypes in D (left), and an additional similar experiment in which the Ras pathway was activated by expression of a *UAS-Raf*^*gof*^ transgene (right). Data are presented as box plots (25%, median and 75% values) with error bars indicating the min and max values.

Brf1 is a conserved component of TFIIIB complex, which is required for Pol III recruitment to tRNA genes [42]. We previously showed that Brf1 is involved in controlling Pol III-dependent transcription, and tissue and body growth in *Drosophila* larvae [39]. Here we examined whether Brf1 is required for Ras-induced tRNA synthesis. We found that knocking down Brf1 using dsRNA in S2 cells (S2A and S2B Fig) suppressed the Ras^V12^ induced increase in tRNA levels (Fig 3A) without altering the strong induction of ERK phosphorylation seen with Ras^V12^ expression (S2B Fig). Hence, our data here suggest that the elevation of tRNA levels upon Ras activation is due to increased Pol III transcription. We then examined whether Brf1 is required for Ras-induced growth in *Drosophila* wing discs. We expressed UAS-driven transgenes in the dorsal compartment of the wing imaginal disc (using an *apterous-Gal4* driver, *ap-GAL4*) and then, in each case, measured tissue size in wandering stage third instar larvae, Overexpression of *UAS-EGFR* (*UAS-λtop*) in the dorsal compartment of the wing imaginal disc stimulates Ras/ERK signalling and leads to tissue growth (Fig 3B and 3C). We found that RNAi-mediated knockdown of Brf1 by expression of a *UAS-Brf1* inverted repeat line (*UAS-Brf1 RNAi*) in the dorsal compartment had little effect on tissue growth. However, expression of *UAS-Brf1 RNAi* blocked the overgrowth seen with *UAS-EGFR* expression. Expression of *UAS-Brf1 RNAi* with *ap-*GAL4 had little effect on tissue growth, suggesting we are not knocking down Brf1 to a level that cannot support any growth. We previously showed that Brf1 knockdown had no effect on ribosome synthesis, suggesting that its predominant effect was to block Pol III function [39]. Hence, these data indicate that Brf1 and Pol III transcription is required for EGFR/Ras/ERK-mediated increases in epithelial tissue growth in *Drosophila*.

### Brf1 is required for Ras/ERK-induced proliferation in adult mid-gut progenitor cells (AMPs) and adult intestinal stem cells (ISCs)

A major role for the EGFR/Ras/ERK pathway is in the growth and maintenance of the *Drosophila* intestine. In larvae, activation of the pathway plays a central role in controlling the proliferation of adult midgut progenitor cells (AMPs), which eventually give rise to the adult intestine [9]. In the adult the EGFR/Ras/ERK pathway is required to promote stem cell proliferation and tissue regeneration [15–17,19,43]. We therefore examined whether Brf1-mediated Pol III transcription was required for these proliferative effects of Ras/ERK signalling. We first examined the larval intestine. During the larval period, AMPs proliferate and give rise to clusters of ~5–10 cells scattered throughout the larval intestine. These cell clusters eventually proliferate and fuse during metamorphosis to give rise to the adult intestinal epithelium. The EGFR/Ras/ERK pathway controls the proliferation of AMPS [9]. Overexpression of either *UAS-EGFR* or *UAS-Raf*^*gof*^ in the AMPs using the temperature-sensitive *escargot-Gal4* (*esg-Gal4*^*ts*^) system lead to a massive increase AMP proliferation and an increase in the numbers of AMP cells per cluster as previously reported. We found that expression of *UAS-Brf1 RNAi* (Fig 3D and 3E and S2C Fig) lead to a small reduction in the number of cells per cluster. However, we found that when co-expressed along with *UAS-EGFR* or *UAS-Raf*^*gof*^, *UAS-Brf1 RNAi* blocked the increase in AMP cell numbers. These data indicate Brf1 is required for EGFR/Ras/ERK mediated cell proliferation.

**Fig 4:**
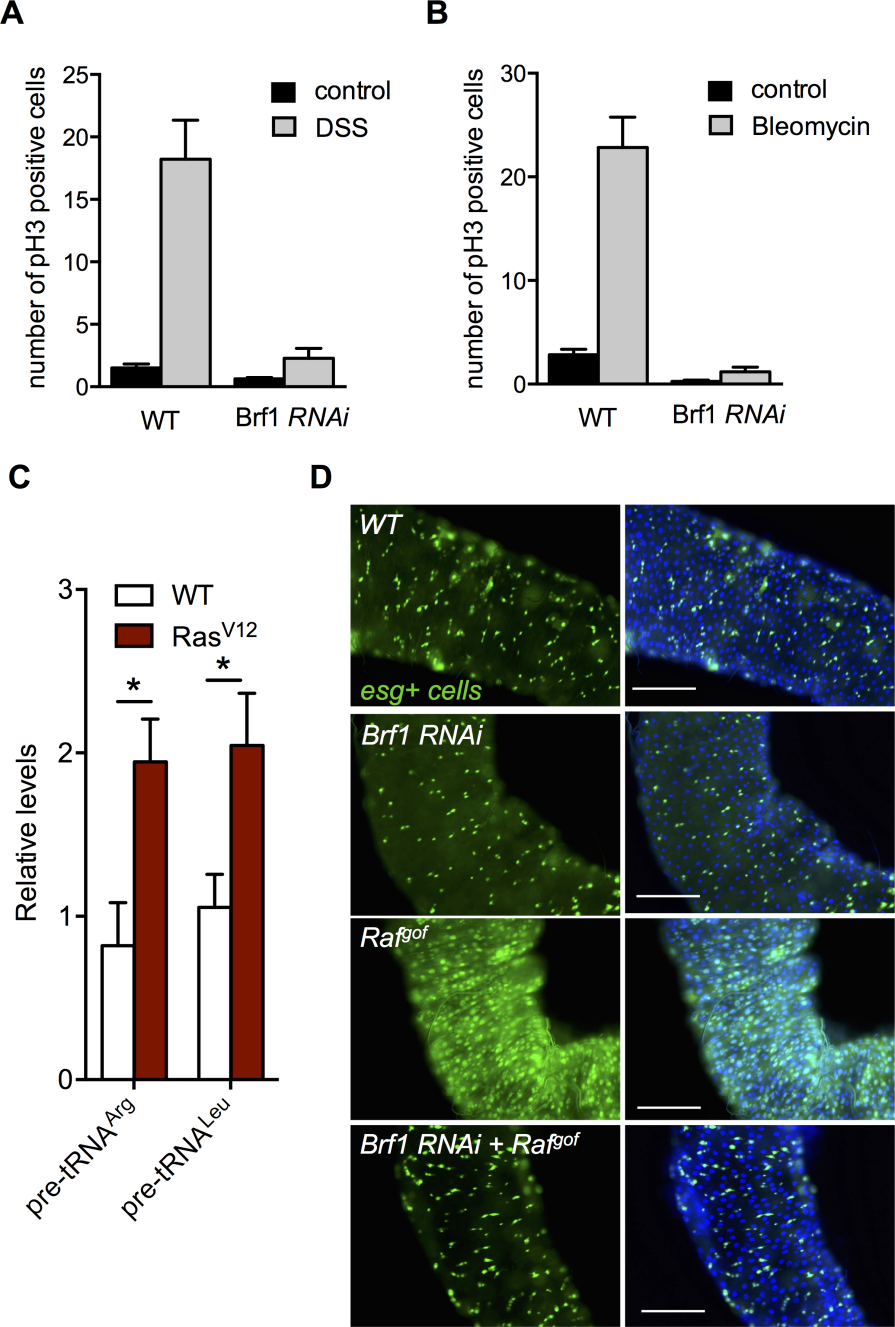
Brf1 is required for intestinal stem cells (ISCs) homeostasis and for Ras-induced cell proliferation. (A, B) *UAS-Brf1 RNAi* was expressed adult ISCs and EBs using the *esg-GAL4*^*ts*^ system. Control flies were *esg-GAL4*^*ts*^ flies crossed to *w*^*1118*^. Flies were then fed with sucrose or sucrose plus DSS (A) or Bleomycin (B) for 2 days. Intestines were then dissected and stained for phospho-histone H3 positive cells. Data represent the mean number of phospho-histone H3 cells per intestine +/ SEM. N >15 intestines per condition. (C) A UAS-Ras^V12^ transgene was expressed in adult intestines using the *esg-GAL4*^*ts*^ driver. Control samples (WT) expressed *UAS-GFP* alone. Total RNA was isolated and levels of pre-tRNAs measured by qRT-PCR. N = 4 independent samples per condition. Data are presented as mean +/- SEM. (D) *UAS-Raf*^*gof*^ and *UAS-Brf1 RNAi* were expressed, either alone or together, in the adult ISCs and EBs using the *esg-Gal4*^*ts*^ system. *esg* positive cells are marked with GFP and DNA is stained with Hoechst dye. Knockdown of Brf 1(*UAS-Brf RNAi*) suppresses the increased proliferation seen with *UAS-Raf*^*gof*^ expression.

We next examined Brf1 function in homeostatic growth in the adult intestine. Damage to intestinal epithelial cells leads to an increase in expression and release of EGF ligands from both intestinal cells and underlying visceral muscle [15]. These EGF ligands then act on the intestinal stem cells (ISCs) to stimulate the Ras/ERK pathway, which triggers stem cell growth and division, and promotes regeneration of the intestinal epithelium. This damage-induced increase in ISC proliferation is dependent on EGFR/Ras/ERK signalling and can be mimicked by genetically activating the pathway specifically in the stem cells [15–17,19]. We tested a requirement for Brf1 in this Ras-mediated homeostatic growth response. We first examined the effects of intestinal damage. As previously reported [19], we found that feeding flies either DSS or bleomycin–two different gut stressors–leads to an increase in ISC proliferation. However, we found that this effect was inhibited when we knocked down Brf1 (using *UAS-Brf1 RNAi* expression) specifically in the ISCs and their transient daughter cells, the enteroblasts (EBs), using the inducible *esg-Gal4*^*ts*^ system (Fig 4A and 4B). We next examined the effects of activation of the Ras/ERK pathway. We first overexpressed *UAS-Ras*^*V12*^ in the adult intestine using the inducible *esg-Gal4*^*ts*^ system, and observed an increase in pre-tRNA levels (Fig 4C). As previously reported, when we overactivated the pathway in stem cells by expressing *UAS-Raf*^*gof*^ using *esg-Gal4*^*ts*^, we saw an increase cell proliferation as indicated by a marked increase in GFP labelled ISCs and EBs (Fig 4D). Expression of a *UAS-Brf1 RNAi* had little effect on GFP labelled cells, but when co-expressed with *UAS-Raf*^*gof*^ it blocked the increase in cell proliferation. These results suggest that Brf1 and Pol III-dependent transcription is required for stem cell proliferation in the adult intestine.

### dMyc is required but not sufficient for Ras-induced tRNA synthesis

**Fig 5:**
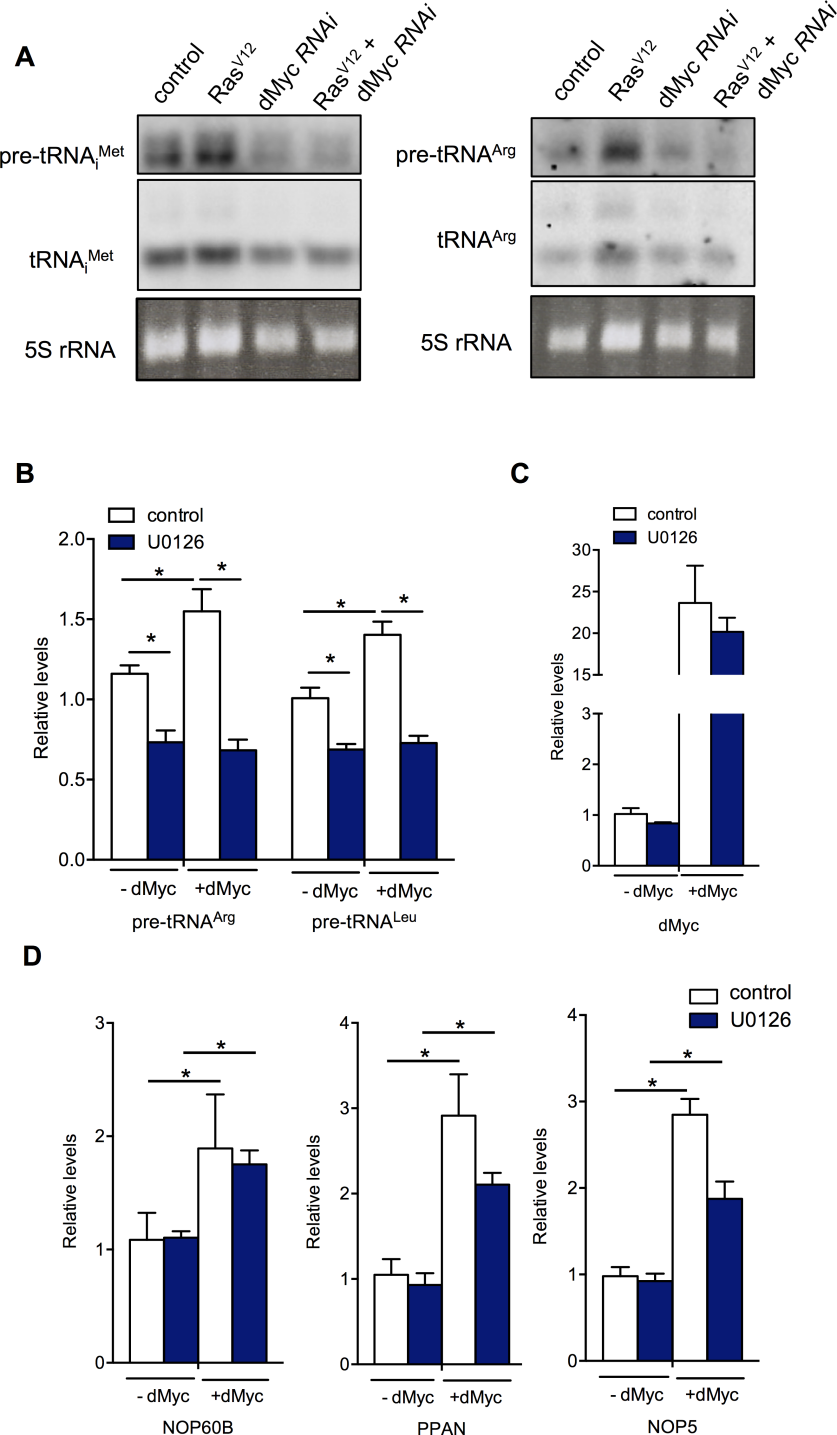
dMyc is required but not sufficient for Ras-induced tRNA synthesis. (A) Ras^V12^ expression was induced in *Drosophila* S2 cells for 24 hours in either control cells or dMyc knockdown cells. dMyc was knocked down by incubating cells with dsRNA against *dMyc*. Control cells were treated with dsRNA to GFP. Total RNA was isolated with Trizol and analyzed by northern blotting using DIG-labelled antisense probes to tRNA^iMet^ or tRNA^Arg^. Ethidium bromide stained 5S rRNA band was used as a loading control. (B, C and D) dMyc expression was induced in S2 cells for 24hrs, and then cells were treated with 10 μM U0126 or DMSO for 2 hours. Total RNA was isolated with Trizol and analyzed by qRT-PCR to measure levels of (B) pre-tRNAs, (C) *dMyc* mRNA, or (D) mRNA levels of three dMyc target genes—*NOP60B*, *PPAN* and *NOP5*. N = 4 independent samples per condition. Data are presented as mean +/-SEM.

We next wanted to examine how Ras signalling stimulates Pol III-dependent tRNA transcription. One candidate regulator we tested was dMyc. In both mammalian cells and *Drosophila*, Myc can interact with Brf1 and stimulate Pol III-dependent transcription [39,44,45]. Moreover, studies in both mammalian cells and *Drosophila* suggest Ras signalling can regulate dMyc levels and that Myc is required for Ras-induced growth[10,23,24,46,47]. Indeed, we found that the *UAS-EGFR-* and *UAS-Ras*^*V12S35*^-induced proliferation of larval AMPs was blocked when we knocked down dMyc by expression of a *UAS-dMyc RNAi* construct (S3A–S3C Fig). We therefore examined whether dMyc functions downstream of Ras in the control of Pol III. Using S2 cells we found that the increase in tRNA levels seen following Ras^V12^ expression was blocked when cells were treated with dsRNA to knockdown dMyc (Fig 5A). In contrast, we found that overexpression of dMyc in S2 cells was not able to induce tRNA synthesis when the Ras pathway was inhibited by treatment with the MEK inhibitor UO126 (Fig 5B). Under these conditions of Ras pathway inhibition, however, dMyc mRNA levels were not affected (Fig 5C) and overexpressed dMyc was still able to significantly stimulate expression of Nop60B, PPAN and NOP5—three dMyc Pol II target genes (Fig 5D)–although the effect on PPAN and NOP5 was somewhat reduced. Nevertheless, these data suggest that U0126 does not simply abrogate dMyc’s ability to stimulate transcription of its target genes, and that dMyc is required, but not sufficient, to mediate the effects of Ras signalling on tRNA synthesis. These data suggest that Ras/ERK signalling can use an additional mechanism to control Pol III transcription.

### Ras signalling promotes tRNA synthesis by inhibiting the RNA pol III repressor dMaf1

**Fig 6:**
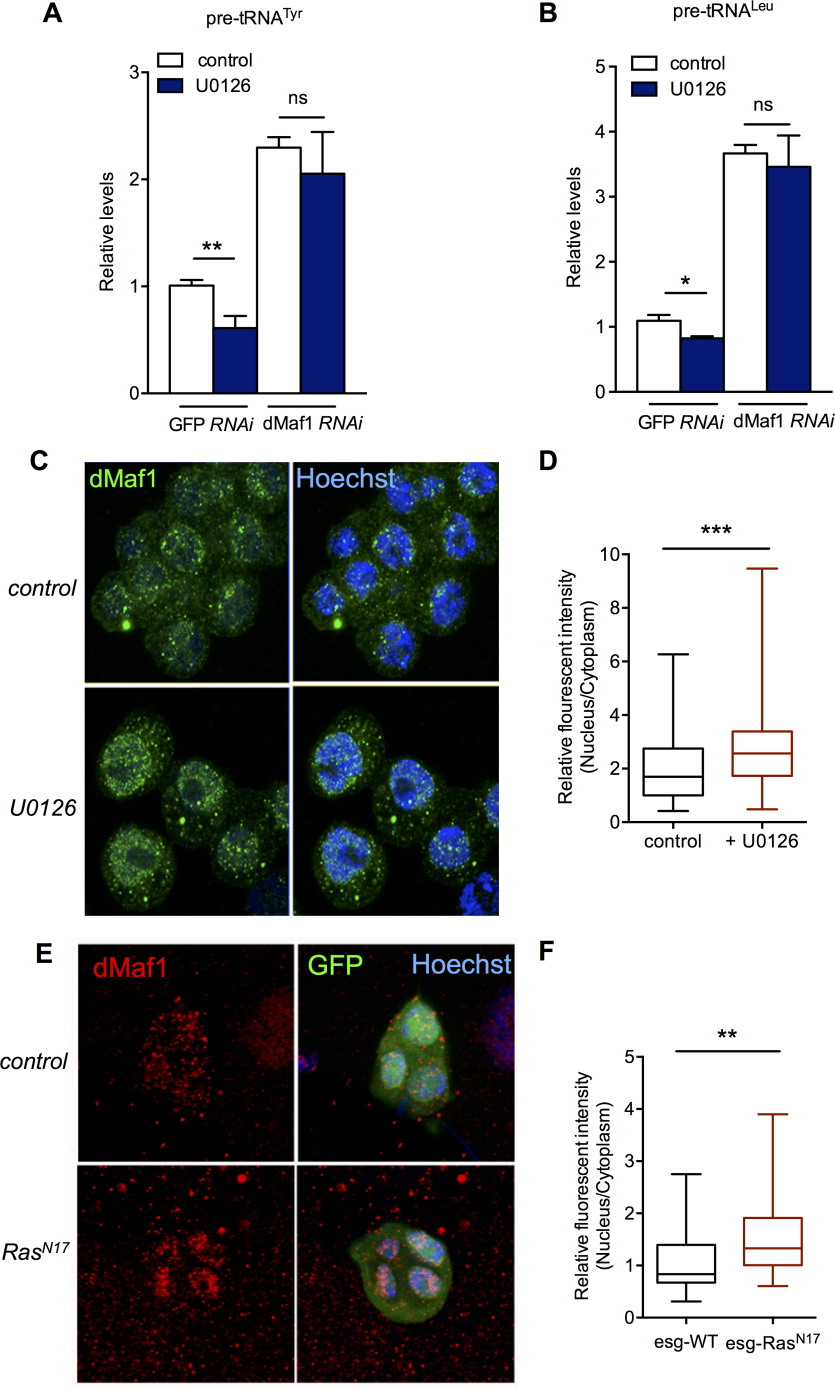
Ras induces tRNA synthesis by via inhibition of the Pol III repressor, dMaf1. (A, B) dMaf1 was knocked down in *Drosophila* S2 cells by incubating cells with dsRNAs against *dMaf1*. Control cells were treated with dsRNA to GFP. Cells were then treated with DMSO (control) or 10 μM U0126 for 2 hrs. Total RNA was isolated and levels of pre-tRNATyr (A) and pre-tRNALeu measured by qRT-PCR. N = 4 independent samples per condition. Data are presented as mean +/-SEM. (C) dMaf1 subcellular localization was assessed by immunostaining with an anti-dMaf1 antibody in both control and 10 μM U0126 treated S2 cells. Green: dMaf1 staining; blue: Hoechst-stained nuclei. (D) The differential localization of dMaf1 in S2 cells from the experiment shown in (C) was quantified by measuring the ratio of nucleus: cytoplasmic intensity quantified using NIH Image J. Data are plotted in the graph as relative nuclear: cytoplasmic staining intensity for both experimental conditions. Data are presented as box plots (25%, median and 75% values) with error bars indicating the min and max values. N>90 cells per condition fom three independent experiments. (E) A *UAS-Ras*^*N17*^ transgene was expressed in GFP-marked adult midgut progenitor cells using the *esg-GAL4*^*ts*^ driver. Control samples (WT) expressed UAS-GFP alone. dMaf1 subcellular localization was then assessed in third instar larvae by immunostaining with an anti-dMaf1 antibody. (F) The differential localization of dMaf1 in the AMPs from the experiment shown in (E) was quantified by measuring the ratio of nucleus: cytoplasmic intensity quantified using NIH Image J. Data are plotted in the graph as relative nuclear: cytoplasmic staining intensity for both experimental conditions. Data are presented as box plots (25%, median and 75% values) with error bars indicating the min and max values. N>30 cells per condition fom three independent experiments.

Another candidate that we considered as a mediator of Ras-induced tRNA synthesis was the conserved Pol III repressor, Maf1. Studies in yeast, *Drosophila* and mammalian cells have shown that inhibition of Maf1 is the main way that the nutrient-dependent TORC1 kinase pathway stimulates Pol III and tRNA synthesis [39,48–51]. Knockdown of *Drosophila* Maf1 (dMaf1) has been shown to promote tRNA synthesis, and to enhance tissue and body growth [40]. Here, we found that when we expressed *UAS-dMaf1 RNAi* in the Ras-responsive AMP cells during larval development using *esg-GAL4*^*ts*^, we observed a modest, but significant increase in the number of AMP cells per cluster (S4A Fig). Although considerably weaker than the effect of Ras pathway activation (e.g. see comparison with effect of *UAS-EGFR*, S4B Fig) this effect of dMaf1 knockdown was similar to the increase in AMP numbers seen with overexpression of dMyc, another stimulator of tRNA synthesis and mRNA translation (S4C Fig). We therefore next examined whether the Ras/ERK pathway functions to promote tRNA synthesis by inhibiting dMaf1. We examined pre-tRNA levels using qRT-PCR in S2 cells, and, as described above, we saw that treatment of cells with the MEK inhibitor UO126 led to reduced tRNA synthesis (Fig 6A and 6B). However, we found that this decrease in tRNA synthesis was reversed when cells were treated with dsRNA to knockdown dMaf1 levels (Fig 6A and 6B). We observed similar effects when we used Northern blotting to measure pre-tRNA and tRNA levels (S4D Fig). We also used treatment of cell with dsRNA to Ras to block Ras signalling, and saw a decrease in tRNA synthesis (S4E Fig). However, as with UO126 treatment, we found that this decrease in tRNA synthesis caused by dsRNA to Ras was reversed by co-treatment of cells with dsRNA to dMaf1. These data suggest that one main way that Ras/Erk signalling functions to promote tRNA synthesis is by inhibiting the Pol III repressor function of dMaf1.

Studies in both yeast and mammals indicate that Maf1can be regulated by controlling its nuclear localization (e.g [50,52]). We first tested this in S2 cells using an antibody to endogenous dMaf1. Under our normal media culture conditions, we observed that dMaf1 was localized throughout the cell (Fig 6C). When we carried out antibody staining in dMaf1 dsRNA-treated cells (which leads to a strong knockdown of both dMaf1 mRNA, S5A Fig, and dMaf1 protein, S5B Fig) we saw minimal background staining, suggesting that the antibody is specific for dMaf1 (S5C Fig). We found that treatment of cells with the MEK inhibitor U0126 lead to a significant increase in nuclear localization of dMaf1 (Fig 6C and 6D), without having any effect on overall dMaf1 protein levels (S5D Fig). We also found that genetic inhibition of Ras signalling in AMPs, by overexpression of dominant-negative Ras (*UAS-Ras^N17^*), lead to an increase in nuclear localization of dMaf1 (Fig 6E and 6F). Similar results were seen when we used expression of either *UAS-EGFR* or *UAS-Ras RNAi* to block Ras signalling in AMPs (Supplemental Fig 6A). Thus, Ras/ERK signalling functions to prevent nuclear accumulation of dMaf1, hence blocking its Pol III repressor activity and promoting tRNA synthesis.

**Fig 7:**
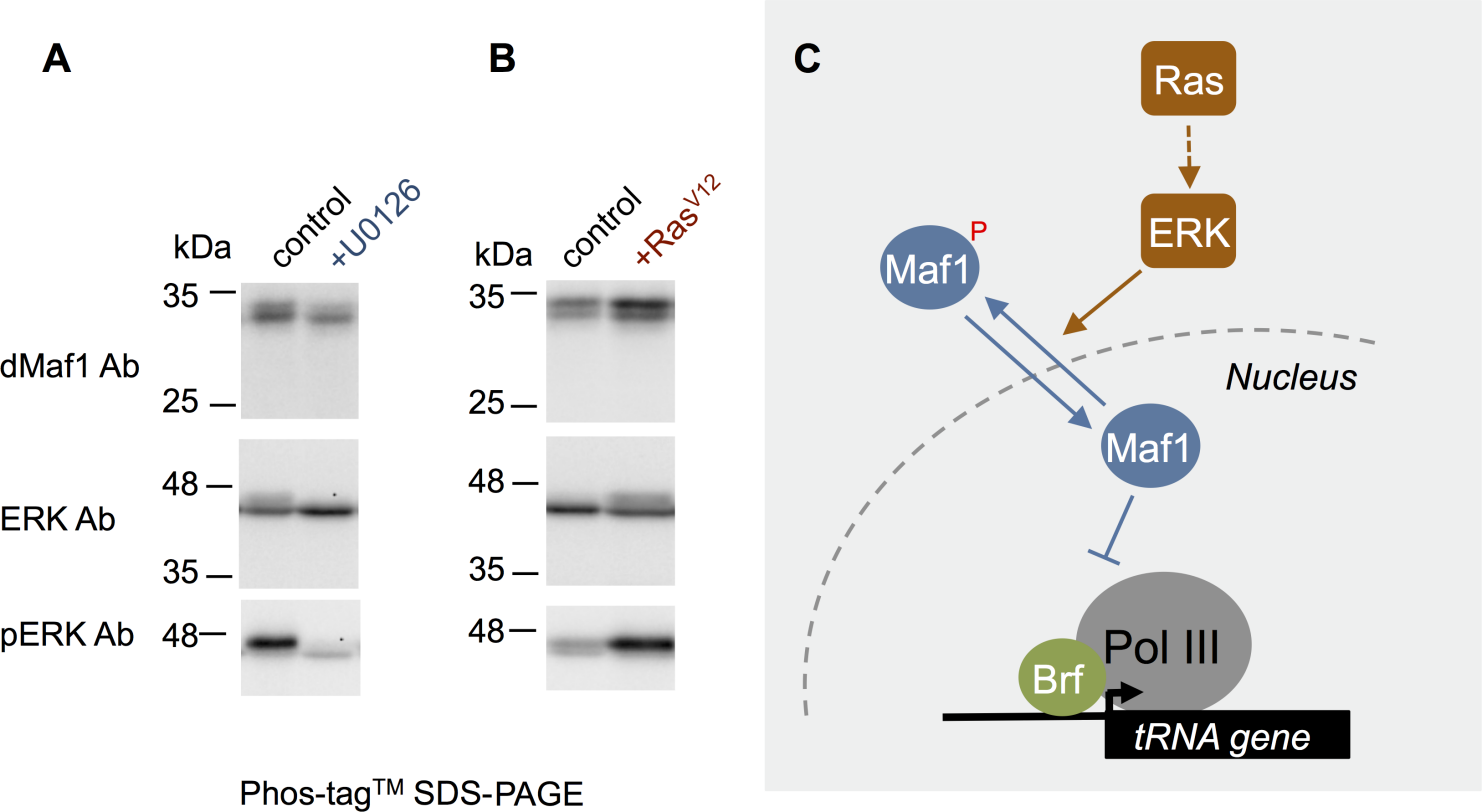
Ras signalling regulates dMaf1 phosphorylation. (A) *Drosophila* S2 cells were treated with 10 μM U0126 for 2 hours. Cells were then lysed and processed for SDS-PAGE and western blotting using the phos-tag reagent, as described in the Methods. The blots were then probed with an anti-dMaf1 antibody (top panel), an anti-total ERK antibody (middle panel) or an anti-phospho ERK antibody (lower panel) (B) Ras^V12^ expression was induced in *Drosophila* S2 cells for 24 hours. Cells were then lysed and processed for SDS-PAGE and western blotting using the phos-tag reagent, as described in the Methods. The blots were then probed with an anti-dMaf1 antibody (top panel), an anti-total ERK antibody (middle panel) or an anti-phospho ERK antibody (lower panel). (C) A model for how Ras signalling may regulate Pol III and tRNA synthesis.

Previous studies showed that the TORC1 pathway can regulate Maf1 nuclear localization and repressor function via phosphorylation [48–51,53]. We therefore explored whether the Ras pathway could also control the phosphorylation status of dMaf1 in S2 cells. We used the phos-tag reagent, which slows the migration of phosphorylated proteins in SDS-PAGE gels, and hence helps resolve phosphorylated vs. non-phosphorylated versions of a protein on a western blot. For example, when we examined total ERK levels by western blotting following SDS-PAGE with Phos-tag, we observed two ERK bands. The relative levels of the upper band were reduced when we treated cells with the MEK inhibitor (Fig 7A), while levels of the upper band were increased in cells overexpressing *Ras*^*V12*^ (Fig 7B), thus indicating this method can detect protein phosphorylation changes. We then examined dMaf1 protein levels in western blots following SDS-PAGE with Phos-tag. As with ERK, we observed two dMaf1 bands, and the relative levels of the upper band were reduced when we treated the sample with phosphatase prior to SDS-PAGE (S6B Fig), suggesting this upper band is a phosphorylated version of dMaf1. Also, like ERK, we found that relative levels of the upper band were reduced when we treated cells with the MEK inhibitor (Fig 7A), while levels of the upper band were increased in cells overexpressing *Ras*^*V12*^ (Fig 7B). Together these data suggest that Ras signalling may regulate dMaf1 phosphorylation, and based on previous work with TORC1 signalling, this may be one way that Ras regulates dMaf1 nuclear vs. cytoplasmic localization (Fig 7C).

## Discussion

We propose that stimulation of RNA polymerase III and tRNA synthesis contributes to the ability of the conserved Ras/ERK pathway to promotes mRNA translation and growth. Our data indicate that Ras can control Pol III by inhibiting the Maf1 repressor, in part by preventing its nuclear accumulation. Maf1 is a phospho protein and studies in yeast and mammalian cells have described how phosphorylation can regulate Maf1 nuclear localization. For example, both TORC1 and PKA can phosphorylate Maf1 on several conserved residues[48–53]. This phosphorylation prevents Maf1 nuclear accumulation and allows both kinases to stimulate Pol III. In contrast, dephosphorylation of Maf1 by both PP2A and PP4 protein phosphatases leads to nuclear accumulation of Maf1 and Pol III repression [54–56]. Thus, it is possible that ERK may function by promoting Maf1 phosphorylation–either directly or indirectly–to prevent its function. Other mechanisms may also be important for Ras to simulate tRNA synthesis. For example, one study in mammalian cells showed that ERK could phosphorylate and regulate Brf1 function [57]. Also, Ras was shown to upregulate TBP, which can increase transcription by all three RNA polymerases [41], although we did not see a similar effect. Interestingly, we found that the decrease in tRNA synthesis caused by inhibiting Ras signalling could be completely reversed by dMaf1 knockdown. This result suggests that while Ras signalling may exert multiple effects to control Pol III transcription, inhibition of dMaf1 seems to be an important effector of Ras in the control of tRNA synthesis. Maf1 function is conserved suggesting that the Ras/ERK-dependent regulation of Maf1 and tRNA synthesis that we describe in *Drosophila* may operate in other organisms, particularly human cells.

Our data using the phos-tag reagent suggest that one way that Ras/ERK signalling may control dMaf1 is via phosphorylation. Previous studies in both yeast and mammalian cells have shown that the TORC1 pathway can control the nuclear localization and repressor function of dMaf1 via phosphorylation of several conserved residues [48–50,53]. One can therefore speculate that Ras signalling may work in a similar manner. Although further studies are required to identify if ERK directly phosphorylates dMaf1 and to identify the phosphorylated residues, it is interesting to note that two of the conserved TORC1 phosphorylation sites on dMaf1 are serine residues followed by proline, which are sites that are often phosphorylated by ERK, a proline-directed kinase.

We also show that the transcription factor dMyc is required for the effects of Ras on Pol III and tRNA synthesis. Previous work from both mammalian cells and *Drosophila* has shown that in some cells Ras can promote Myc levels and that Ras-mediated growth requires Myc function[10,23,24,46,47]. We previously showed that *Drosophila* Myc could stimulate expression of the Pol III transcription factor, Brf1, and also other Pol III subunits [39]. In addition, Myc can directly interact with Brf1 and localize at Pol III to directly stimulate tRNA transcription in Drosophila and mammalian cells [39,44,45]. We suggest that both these effects are under the upstream control of Ras/ERK signalling and may, in part, explain the requirements for Myc in Ras-induced growth in both animal development and cancer.

Given our findings with dMaf1 and dMyc, we attempted to address which of the two mechanisms—inhibition of dMaf1 or activation of Myc—might explain the main effects of Ras/ERK signalling on tRNA synthesis. To do this, we inhibited Ras/ERK signalling in S2 cells and then asked whether knockdown of dMaf1 or overexpression of dMyc could maintain tRNA synthesis. We found that, of these two manipulations, only dMaf1 inhibition could restore tRNA synthesis when ERK signalling was inhibited. We interpret these findings to suggest that, while dMyc is required for tRNA synthesis, it is the inhibition of dMaf1 that explains a substantial part of the mechanism of action of Ras/ERK signalling in the regulation of Pol III and tRNA synthesis. We previously showed that dMaf1 knockdown does not alter expression of dMyc target genes [39], suggesting that enhancement of dMyc function doesn’t explain why Maf1 knockdown can maintain tRNA synthesis in cells in which Ras/ERK signalling is inhibited.

Previous studies in mammalian cells have shown that Ras/ERK signalling can promote protein synthesis by stimulating translation initiation factor function. We suggest that inhibition of Maf1 represents another target of Ras/ERK signalling, and that the subsequent increase in tRNA levels may cooperate with enhanced translation initiation factor activity to promote maximal stimulation of mRNA translation. Most of the work on Ras-mediated gene expression has focused on the effect of several Pol II transcription factors identified downstream of Ras in *Drosophila* such as *fos*, *pointed*, and *capicua* [19–22]. Stimulation of Pol III transcription to enhance tRNA levels and mRNA translation may provide another layer of control on overall gene expression by Ras signalling. For example, translational control of cell cycle genes has been proposed as one way to couple growth signalling pathways to cellular proliferation [58,59]. Furthermore, selective translational regulation of certain mRNAs has been shown to regulate growth and metastatic behaviour of tumour cells [60–62]. It important to note though that we find that simply knocking down dMaf1 alone has only a modest effect on cell proliferation AMPs, compared to the strong hyperproliferative effect of overactivation of Ras signalling. This is likely because increasing Pol III is only one downstream effect of Ras signalling and that the full Ras effect on cell proliferation requires the coordinated increase in the expression of many genes. Indeed, it is likely that Ras stimulates the activity of all three RNA polymerases to drive cell growth and proliferation.

Ras is one of the most often overactivated or mutated pathways in cancer, hence our findings may also have implications for processes that contribute to tumour growth and metastasis. Indeed, there is increasing appreciation for potential roles for alterations in tRNA biology in cancer cells [63]. For example, tRNA expression profiling has revealed that levels of many tRNAs are elevated in different cancer types [64,65]. Interestingly, these changes in tRNA levels have been shown to correlate with codon usage in mRNAs whose expression also changes in cancer cells [66]. Several studies have reported that increasing the levels of specific tRNAs can promote tumour growth and metastatic behavior [67–70]. Previous work also showed that increasing tRNA levels alone is sufficient to drive growth in *Drosophila* [40,71]. Hence, an increase in tRNA levels caused by oncogenic Ras signalling may be a driver of tumour growth and progression, rather than simply a consequence of increased growth. Ras also controls other process such as cell fate specification, differentiation and cell survival. Many of these effects are mediated through translation and so may also rely on the effects of Ras on tRNA synthesis.

## Materials and methods

### *Drosophila* stocks

Flies were raised on standard medium (150 g agar, 1600 g cornmeal, 770 g Torula yeast, 675 g sucrose, 2340 g D-glucose, 240 ml acid mixture (propionic acid/phosphoric acid) per 34 L water) and maintained at 25°C, unless otherwise indicated. The following fly stocks were used:

*w*^*1118*^,
*yw*,
*UAS-Ras*^*V12*^*, [24]*
*UAS-Ras*^*N17*^*, [24]*
*UAS-Ras*^*V12S35*^*, [24]*
UAS-EGFR (UAS-λTOP), [24]
*UAS-Raf*^*gof*^*, [24]*
*UAS-Brf RNAi (NIG*, *Japan)*,
UAS-dMyc [72]
UAS-Maf1 RNAi [40],
*esg-gal4*, *tub-GAL80*^*ts*^*, UAS-GFP, [15]*
*ap-gal4/CyO*,
*dpp-gal4/TM6B*.

For all GAL4/UAS experiments, GAL4 lines were crossed to the relevant UAS line(s) and the larval or adult progeny were analyzed. Control animals were obtained by crossing the relevant GAL4 line to either *w*^*1118*^ or *yw* depending on the genetic background of the particular experimental UAS transgene line. For the *esg-gal4*^*ts*^ system, larvae and were flies were initially raised at 18°C and then for each experiment they were shifted to 29°C to inactivate the temperature sensitive GAL80 and to allow GAL4-mediated transgene expression.

### Cell culture and transfection

*Drosophila* Schneider S2 cells were grown at 25^**°**^C in Schneider’s medium (Gibco; 11720–034) supplemented with 10% fetal bovine serum (Gibco; 10082–139), 100 U/ml penicillin and 100 U/ml streptomycin (Gibco; 15140). Stably transfected inducible Ras^V12^ cells were a gift from the lab of Marc Therrien [73]. Stably transfected inducible dMyc cells were a gift from the lab of Paula Bellosta [74]. Both Ras^V12^ and dMyc expression are under the control of a metallothionein promoter. For all experiments Ras^V12^ or dMyc were induced by addition of copper sulphate to the culture media.

*dsRNA Treatment of S2 cells*: dsRNAs were synthesized with RiboMAX large-scale RNA production system (Promega) using PCR products from either cDNAs or genomic DNA (primer sequences in S2 Table). Cells were pretreated with 15 μg of dsRNAs in the absence of serum for 30 mins and then 2 mls of media plus serum was added, and cells were then incubated for 96 to 120 hrs. Control cells were treated with ds RNA to Green Fluorescent Protein (GFP). Cells were harvested by centrifugation at 4 °C and washed with cold PBS and frozen for RNA isolation or protein extraction.

*MEK inhibitor (U0126) treatment of Drosophila S2 cells*: S2 cells were cultured at 25^**°**^C in Schneider’s medium (Gibco; 11720–034) supplemented with 10% fetal bovine serum (Gibco; 10082–139), 100 U/ml penicillin and 100 U/ml streptomycin (Gibco; 15140). Cells were treated with either 10 μM U0126 (Promega Cat. No. V1121) or DMSO (Sigma; D2650) for 2 hours. Then cells were washed twice with ice-cold PBS. Cells were then used to isolate RNA or make protein extracts as described below.

### Preparation of protein extracts

*Drosophila* S2 cells were lysed with a buffer containing 20 mM Tris-HCl (pH 8.0), 137 mM NaCl, 1 mM EDTA, 25% glycerol, 1% NP-40 and with following inhibitors 50 mM NaF, 1 mM PMSF, 1 mM DTT, 5 mM sodium ortho vanadate (Na_3_VO_4_) and Protease Inhibitor cocktail (Roche Cat. No. 04693124001) and Phosphatase inhibitor (Roche Cat. No. 04906845001) according to the manufacturer’s instruction.

### Phos-tag SDS-PAGE

*Drosophila* S2 cells were lysed with a buffer containing 20 mM Tris-HCl (pH 8.0), 137 mM NaCl, 25% glycerol, 1% NP-40 and with following inhibitors 1 mM PMSF, 1 mM DTT and Protease Inhibitor cocktail (Roche Cat. No. 04693124001) and Phosphatase inhibitor without EDTA. Phos-tag SDS-PAGE was prepared according to the manufacturer’s instruction (Wako Chemicals USA, Inc). Cell lysates were separated on 12.5% SDS-polyacrylamide gel containing 20 uM Phos-tag acrylamide (AAL-107 Wako Chemicals USA, Inc), and transferred onto PVDF membranes (Bio Rad).

### Western blot and antibodies

Protein concentrations were measured using the Bio-Rad Dc Protein Assay kit II (5000112). Protein lysates (15 μg to 30μg) were resolved by SDS–PAGE and electrotransferred to a nitrocellulose membrane, subjected to Western blot analysis with specific antibodies, and visualized by chemiluminescence (enhanced ECL solution (Perkin Elmer). Brf primary antibodies were against a C-terminal fragment of *Drosophila* Brf, alpha-tubulin (E7, *Drosophila* Studies Hybridoma Bank), dMyc [24], phospho-ERK (Cell Signalling Technology 4370) and ERK (Cell Signalling Technology 4695). Peptide antiserum against *Drosophila* Maf1 was raised by immunizing rabbits with synthetic peptide LADFSPNFRC corresponding to residues 65–74 (GL Biochem (Shanghai) Ltd).

### Puromycin-labelling protein synthesis assay

10 μM puromycin was added to *Drosophila* S2 cell culture media and the cells were incubated with puromycin for 30 min at 25 °C. Cells were harvested by centrifugation at 4°C and washed with cold PBS. Cells were frozen on dry ice and then lysed according to the Western blot protocol described above and analyzed by SDS-PAGE and western blotting using an anti-puromycin antibody (3RH11) (Kerafast, Catalog No.EQ0001) at 1:2000 dilution.

### Northern blot analysis

Total RNA was extracted from *Drosophila* S2 cells using TRIzol. 5 μg total RNA was separated on a 5% denaturing polyacrylamide/urea gel and northern blotting was carried using alkaline transfer. Hybridization of tRNA probes were carried out as described in Roche DIG Easy Hyb (Cat. No.11603558001). Digoxigenin-labelled probes were made by *in vitro* transcription using either full-length cDNAs or PCR fragments as templates. Primers used for PCR are included in S1 Table.

### Immunostaining

*Drosophila* S2 cells were fixed in 4% paraformaldehyde at room temperature for 20 mins on cover slips. Cells were then washed with 1x PBS and permeabilized with 0.1% Triton X in PBS by washing 2x for 5 mins. Cells were blocked with 5% FBS, 0.1% Triton X in PBS for 2 hours. Primary dMaf1 antibody was diluted in 5% BSA in PBS at 1:500 dilution and incubated overnight at 4°C. Then washed 3x with 0.1% Triton X in PBS for 5 min each and Alexa 568 (Molecular probes) goat-anti rabbit secondary antibody was diluted at 1:400 in 5% BSA in PBS for 2 hours at room temperature. Then, cells were washed 3x with 0.1% Triton X in PBS for 5 min each and mounted using VectaShield mounting medium.

*Drosophila* larvae were inverted and fixed in 8% paraformaldehyde/PBS at room temperature for 45 mins. After blocking for 2hrs in 1%BSA in PBS/0.1% Triton-X 100, larval carcasses were incubated overnight in anti-dMaf1 antibody (1:1000). Primary antibody staining was detected using Alexa 488 (Molecular probes) goat-anti rabbit secondary antibodies.

For experiments looking at dMaf1 subcellular localization, we used Image J to measure dMaf1 staining intensity. Nuclear localization was measured was calculated by measuring the total intensity of signal in the nucleus and dividing this by the total intensity in the cytoplasm (calculated as total overall cellular signal intensity minus total nuclear signal intensity).

### Real-time quantitative PCR

Total RNA was extracted using TRIzol according to manufacturer’s instructions (Invitrogen; 15596–018). RNA samples were DNase treated according to manufacturer’s instructions (Ambion; 2238G) and reverse transcribed using Superscript II (Invitrogen; 100004925). The generated cDNA was used as a template to perform qRT–PCRs (ABI 7500 real time PCR system using SyBr Green PCR mix) using specific primer pairs (sequences available upon request). PCR data were normalized to either actin or Glyceraldehyde-3-phosphate dehydrogenase (GAPDH) levels. Each experiment was independently repeated a minimum of three times. All primer sequences are in S3 Table.

### Polysome gradient centrifugation

Polysome gradient centrifugation was performed as described [40]. 100 million *Drosophila* S2 cells were lysed in 1 ml of lysis buffer (25 mM Tris pH 7.4, 10 mM MgCl_2_, 250 mM NaCl, 1% Triton X-100, 0.5% sodium deoxycholate, 0.5 mM DTT, 100 mg/ml cycloheximide, 1 mg/ml heparin, Complete mini Roche protease inhibitor (Roche), 2.5 mM PMSF, 5 mM sodium fluoride, 1 mM sodium orthovanadate and 200 U/ml ribolock RNAse inhibitor (Fermentas) using a Dounce homogenizer. The lysates were centrifuged at 15,000 rpm for 20 minutes and the supernatant was removed carefully. 150 to 250 g μg RNA was layered gently on top of a 15–45% w/w sucrose gradient (made using 25 mM Tris pH 7.4, 10 mM MgCl2, 250 mM NaCl, 1 mg/ml heparin, 100 mg/ml cycloheximide in 12 ml polyallomer tube) and centrifuged at 37,000 rpm for 150 minutes in a Beckmann Coulter Optima L-90K ultracentrifuge using a SW-41 rotor. Polysome profiles were obtained by pushing the gradient using 70% w/v Sucrose pumped at 1.5 ml/min into a continuous OD254 nm reader (ISCO UA6 UV detector) showing the OD corresponding to the RNA present from the top to the bottom of the gradient.

### Statistical analysis

All qRT-PCR data and quantification of immunostaining data were analyzed by Students t-test, or two-way ANOVA followed by post-hoc students t-test where appropriate. All statistical analysis and data plots were performed using Prism software. In all figures, statistically significant differences are presented as: * p<0.05, ** p<0.005, *** p<0.0005, **** p<0.0001.

## Supporting information

S1 Fig The Ras/ERK signalling pathway regulates tRNA synthesis (related to Fig 2). (A) Left: S2 cells were treated with (10μM) U0126 for 2 hrs. Total RNA was isolated and analyzed by Northern blot. Levels of tRNA^Arg^ were detected using DIG-labelled tRNA probes. Ethidium bromide stained 5S rRNA bands were used as loading controls, since total 5S rRNA levels are unchanged by manipulations in Ras/Erk signalling (see C). (B) Ras^V12^ expression was induced in S2 cells for 24hrs. Total RNA was isolated and analyzed by northern blot. Levels of tRNA^Arg^ were detected using DIG-labelled tRNA probes. Ethidium bromide stained 5S rRNA bands were used as loading controls, since total 5S rRNA levels are unchanged by manipulations in Ras/Erk signalling (see C) (C) qRT-PCR measurement of Brf1 mRNA, Trf1 mRNA, TBP mRNA, 5S rRNA and 7SL RNA levels in U0126 treated S2 cells. (D) qRT-PCR measurement of 5S rRNA levels in Ras^V12^ overexpressing S2 cells.
(TIFF)

S2 Fig Brf1 is required for Ras-induced cell proliferation in AMPs (related to Fig 3). (A) Brf1 mRNA levels were measured by qRT-PCR in *Drosophila* S2 cells treated with dsRNA against Brf1 or GFP (control). Control cells were treated with GFP dsRNA (B) Brf1, phospho-ERK levels and alpha-tubulin protein levels were measured by western blot in *Drosophila* S2 cells treated with dsRNA against Brf1 and overexpressing RasV12, both alone and together. (C) *UAS-Raf*^*gof*^ and *UAS-Brf1 RNAi* were expressed, either alone or together, in the Drosophila larval AMPs using the *esg-Gal4*^*ts*^ system. Larvae were shifted to 29°C at 24 hrs of development to induce transgene expression and dissected as L3 larvae. AMPs are marked *by UAS-GFP* expression. DNA is stained with Hoechst dye (blue) Representative images are shown for each genotype.
(TIFF)

S3 Fig dMyc is required for Ras-induced AMP cell proliferation (related to Fig 5). *UAS-EGFR* (A) or *UAS-Ras*^*V12S35*^ (C) were expressed, either alone or together with *UAS-dMyc RNAi* in the *Drosophila* larval AMPs using the *esg-Gal4*^*ts*^ system. Larvae were shifted to 29°C at 24 hrs of development to induce transgene expression and dissected as L3 larvae. AMPs are marked by *UAS-GFP* expression. DNA is stained with Hoechst dye (blue) (B) (related to experiment in A) Numbers of cells in each AMP cluster were counted and expressed as box plots.
(TIFF)

S4 Fig Ras-functions via dMaf1 inhibition (related to Fig 6). (A, B) *UAS-dMaf1 RNAi* (A), *UAS-EGFR* (B), or *UAS-dMyc* (C) were expressed in AMPs using the *esg-Gal4*^*ts*^ system. Larvae were shifted to 29°C at 24hrs of development and dissected at wandering stage. The numbers of cells in each AMP cluster were counted and expressed in box plots. (D) dMaf1 and Ras were knocked down, both alone and together, in *Drosophila* S2 cells by incubating cells with dsRNAs against dMaf1 and Ras. Control cells were treated with dsRNA to GFP. Total RNA was isolated with Trizol and analyzed by Northern blot using DIG-labelled tRNA^Arg^ probe. Ethidium bromide stained 5S rRNA band was used as a loading control.
(TIFF)

S5 Fig Effect of Ras signalling on dMaf1 levels (related to Fig 6). (A, B) dMaf1 mRNA levels (A) or protein levels (B) were measured by qRT-PCR or Western blot respectively in cells treated with dsRNA to GFP (control) or dMaf1 (dMaf1 RNAi). dsRNA treatment produced a strong knockdown of dMaf1 levels. (C) Control and dMaf1 dsRNA-treated S2 cells were stained with an anti-dMaf1 antibody (green) and Hoechst dye (blue). (D) dMaf1and Brf1 protein levels were analyzed with western blotting after treatment with U0126 for 2 hours. Decreased phospho-ERK levels served as a positive control for UO126-mediated MEK inhibition. Tubulin levels served as a loading control.
(TIFF)

S6 Fig dMaf1 localizes to the nucleus upon inhibition of the Ras signalling pathway (related to Fig 6). *WT*, *UAS-EGFR RNAi*, *UAS-Ras RNAi* were expressed in AMPs using the *esg-Gal4*^*ts*^ system. Larvae were shifted to 29°C at 24hrs of development and dissected at wandering stage and stained with *dMaf1* antibody. (B) *Drosophila* S2 cell lysates (left, control samples; right, Ras^V12^ induced samples) were treated with either Alkaline phosphatase or λ-phosphatase for 1 hr at 37°C and samples were analysed by phos-tag SDS-PAGE and western blotting using an anti-dMaf1 antibody.
(TIFF)

S1 Table List of sequence for Northern probe synthesis. (TIFF)

S2 Table List of primers for dsRNA. (TIFF)

S3 Table List of sequence for qRT-PCR. (TIFF)

## References

[1] Pylayeva-Gupta Y, Grabocka E, Bar-Sagi D (2011) RAS oncogenes: weaving a tumorigenic web. Nat Rev Cancer 11: 761–774. doi: 10.1038/nrc3106 21993244 10.1038/nrc3106 PMC3632399

[2] Rauen KA (2013) The RASopathies. Annu Rev Genomics Hum Genet 14: 355–369. doi: 10.1146/annurev-genom-091212-153523 23875798 10.1146/annurev-genom-091212-153523 PMC4115674

[3] Rubin GM, Chang HC, Karim F, Laverty T, Michaud NR, et al (1997) Signal transduction downstream from Ras in Drosophila. Cold Spring Harb Symp Quant Biol 62: 347–352. 9598368

[4] Wassarman DA, Therrien M, Rubin GM (1995) The Ras signalling pathway in Drosophila. Curr Opin Genet Dev 5: 44–50. 7749324 10.1016/s0959-437x(95)90052-7

[5] Ninov N, Manjon C, Martin-Blanco E (2009) Dynamic control of cell cycle and growth coupling by ecdysone, EGFR, and PI3K signalling in Drosophila histoblasts. PLoS Biol 7: e1000079 doi: 10.1371/journal.pbio.1000079 19355788 10.1371/journal.pbio.1000079 PMC2672598

[6] Asha H, Nagy I, Kovacs G, Stetson D, Ando I, et al (2003) Analysis of Ras-induced overproliferation in Drosophila hemocytes. Genetics 163: 203–215. 12586708 10.1093/genetics/163.1.203 PMC1462399

[7] Read RD, Cavenee WK, Furnari FB, Thomas JB (2009) A drosophila model for EGFR-Ras and PI3K-dependent human glioma. PLoS Genet 5: e1000374 doi: 10.1371/journal.pgen.1000374 19214224 10.1371/journal.pgen.1000374 PMC2636203

[8] Parker J (2006) Control of compartment size by an EGF ligand from neighboring cells. Curr Biol 16: 2058–2065. doi: 10.1016/j.cub.2006.08.092 17055987 10.1016/j.cub.2006.08.092

[9] Jiang H, Edgar BA (2009) EGFR signalling regulates the proliferation of Drosophila adult midgut progenitors. Development 136: 483–493. doi: 10.1242/dev.026955 19141677 10.1242/dev.026955 PMC2687592

[10] Prober DA, Edgar BA (2000) Ras1 promotes cellular growth in the Drosophila wing. Cell 100: 435–446. 10693760 10.1016/s0092-8674(00)80679-0

[11] Karim FD, Rubin GM (1998) Ectopic expression of activated Ras1 induces hyperplastic growth and increased cell death in Drosophila imaginal tissues. Development 125: 1–9. 9389658 10.1242/dev.125.1.1

[12] Zecca M, Struhl G (2002) Control of growth and patterning of the Drosophila wing imaginal disc by EGFR-mediated signalling. Development 129: 1369–1376. 11880346 10.1242/dev.129.6.1369

[13] Nagaraj R, Pickup AT, Howes R, Moses K, Freeman M, et al (1999) Role of the EGF receptor pathway in growth and patterning of the Drosophila wing through the regulation of vestigial. Development 126: 975–985. 9927598 10.1242/dev.126.5.975

[14] Halfar K, Rommel C, Stocker H, Hafen E (2001) Ras controls growth, survival and differentiation in the Drosophila eye by different thresholds of MAP kinase activity. Development 128: 1687–1696. 11290305 10.1242/dev.128.9.1687

[15] Jiang H, Grenley MO, Bravo MJ, Blumhagen RZ, Edgar BA (2011) EGFR/Ras/MAPK signalling mediates adult midgut epithelial homeostasis and regeneration in Drosophila. Cell Stem Cell 8: 84–95. doi: 10.1016/j.stem.2010.11.026 21167805 10.1016/j.stem.2010.11.026 PMC3021119

[16] Xu N, Wang SQ, Tan D, Gao Y, Lin G, et al (2011) EGFR, Wingless and JAK/STAT signalling cooperatively maintain Drosophila intestinal stem cells. Dev Biol 354: 31–43. doi: 10.1016/j.ydbio.2011.03.018 21440535 10.1016/j.ydbio.2011.03.018

[17] Buchon N, Broderick NA, Kuraishi T, Lemaitre B (2010) Drosophila EGFR pathway coordinates stem cell proliferation and gut remodeling following infection. BMC Biol 8: 152 doi: 10.1186/1741-7007-8-152 21176204 10.1186/1741-7007-8-152 PMC3022776

[18] Castanieto A, Johnston MJ, Nystul TG (2014) EGFR signalling promotes self-renewal through the establishment of cell polarity in Drosophila follicle stem cells. Elife 3. 10.7554/eLife.04437 PMC4298699 25437306

[19] Biteau B, Jasper H (2011) EGF signalling regulates the proliferation of intestinal stem cells in Drosophila. Development 138: 1045–1055. doi: 10.1242/dev.056671 21307097 10.1242/dev.056671 PMC3042864

[20] Jin Y, Ha N, Fores M, Xiang J, Glasser C, et al (2015) EGFR/Ras Signalling Controls Drosophila Intestinal Stem Cell Proliferation via Capicua-Regulated Genes. PLoS Genet 11: e1005634 doi: 10.1371/journal.pgen.1005634 26683696 10.1371/journal.pgen.1005634 PMC4684324

[21] Baonza A, Murawsky CM, Travers AA, Freeman M (2002) Pointed and Tramtrack69 establish an EGFR-dependent transcriptional switch to regulate mitosis. Nat Cell Biol 4: 976–980. doi: 10.1038/ncb887 12447387 10.1038/ncb887

[22] Tseng AS, Tapon N, Kanda H, Cigizoglu S, Edelmann L, et al (2007) Capicua regulates cell proliferation downstream of the receptor tyrosine kinase/ras signalling pathway. Curr Biol 17: 728–733. doi: 10.1016/j.cub.2007.03.023 17398096 10.1016/j.cub.2007.03.023 PMC2699483

[23] Ren F, Shi Q, Chen Y, Jiang A, Ip YT, et al (2013) Drosophila Myc integrates multiple signalling pathways to regulate intestinal stem cell proliferation during midgut regeneration. Cell Res 23: 1133–1146. doi: 10.1038/cr.2013.101 23896988 10.1038/cr.2013.101 PMC3760623

[24] Prober DA, Edgar BA (2002) Interactions between Ras1, dMyc, and dPI3K signalling in the developing Drosophila wing. Genes Dev 16: 2286–2299. doi: 10.1101/gad.991102 12208851 10.1101/gad.991102 PMC186666

[25] Herranz H, Hong X, Cohen SM (2012) Mutual repression by bantam miRNA and Capicua links the EGFR/MAPK and Hippo pathways in growth control. Curr Biol 22: 651–657. doi: 10.1016/j.cub.2012.02.050 22445297 10.1016/j.cub.2012.02.050

[26] Herranz H, Hong X, Hung NT, Voorhoeve PM, Cohen SM (2012) Oncogenic cooperation between SOCS family proteins and EGFR identified using a Drosophila epithelial transformation model. Genes Dev 26: 1602–1611. doi: 10.1101/gad.192021.112 22802531 10.1101/gad.192021.112 PMC3404387

[27] Reddy BV, Irvine KD (2013) Regulation of Hippo signalling by EGFR-MAPK signalling through Ajuba family proteins. Dev Cell 24: 459–471. doi: 10.1016/j.devcel.2013.01.020 23484853 10.1016/j.devcel.2013.01.020 PMC3624988

[28] Roux PP, Topisirovic I (2012) Regulation of mRNA translation by signalling pathways. Cold Spring Harb Perspect Biol 4. 10.1101/cshperspect.a012252 PMC3536343 22888049

[29] Waskiewicz AJ, Johnson JC, Penn B, Mahalingam M, Kimball SR, et al (1999) Phosphorylation of the cap-binding protein eukaryotic translation initiation factor 4E by protein kinase Mnk1 in vivo. Mol Cell Biol 19: 1871–1880. 10022874 10.1128/mcb.19.3.1871 PMC83980

[30] Roux PP, Shahbazian D, Vu H, Holz MK, Cohen MS, et al (2007) RAS/ERK signalling promotes site-specific ribosomal protein S6 phosphorylation via RSK and stimulates cap-dependent translation. J Biol Chem 282: 14056–14064. doi: 10.1074/jbc.M700906200 17360704 10.1074/jbc.M700906200 PMC3618456

[31] Romeo Y, Moreau J, Zindy PJ, Saba-El-Leil M, Lavoie G, et al (2013) RSK regulates activated BRAF signalling to mTORC1 and promotes melanoma growth. Oncogene 32: 2917–2926. doi: 10.1038/onc.2012.312 22797077 10.1038/onc.2012.312 PMC4440665

[32] Romeo Y, Roux PP (2011) Paving the way for targeting RSK in cancer. Expert Opin Ther Targets 15: 5–9. doi: 10.1517/14728222.2010.531014 20958120 10.1517/14728222.2010.531014

[33] Ueda T, Watanabe-Fukunaga R, Fukuyama H, Nagata S, Fukunaga R (2004) Mnk2 and Mnk1 are essential for constitutive and inducible phosphorylation of eukaryotic initiation factor 4E but not for cell growth or development. Mol Cell Biol 24: 6539–6549. doi: 10.1128/MCB.24.15.6539-6549.2004 15254222 10.1128/MCB.24.15.6539-6549.2004 PMC444855

[34] Ueda T, Sasaki M, Elia AJ, Chio II, Hamada K, et al (2010) Combined deficiency for MAP kinase-interacting kinase 1 and 2 (Mnk1 and Mnk2) delays tumor development. Proc Natl Acad Sci U S A 107: 13984–13990. doi: 10.1073/pnas.1008136107 20679220 10.1073/pnas.1008136107 PMC2922567

[35] Dumont J, Umbhauer M, Rassinier P, Hanauer A, Verlhac MH (2005) p90Rsk is not involved in cytostatic factor arrest in mouse oocytes. J Cell Biol 169: 227–231. doi: 10.1083/jcb.200501027 15837801 10.1083/jcb.200501027 PMC2171868

[36] Arquier N, Bourouis M, Colombani J, Leopold P (2005) Drosophila Lk6 kinase controls phosphorylation of eukaryotic translation initiation factor 4E and promotes normal growth and development. Curr Biol 15: 19–23. doi: 10.1016/j.cub.2004.12.037 15649359 10.1016/j.cub.2004.12.037

[37] Reiling JH, Doepfner KT, Hafen E, Stocker H (2005) Diet-dependent effects of the Drosophila Mnk1/Mnk2 homolog Lk6 on growth via eIF4E. Curr Biol 15: 24–30. doi: 10.1016/j.cub.2004.12.034 15649360 10.1016/j.cub.2004.12.034

[38] Deliu LP, Ghosh A, Grewal SS (2017) Investigation of protein synthesis in Drosophila larvae using puromycin labelling. Biol Open 6: 1229–1234. doi: 10.1242/bio.026294 28642244 10.1242/bio.026294 PMC5576084

[39] Marshall L, Rideout EJ, Grewal SS (2012) Nutrient/TOR-dependent regulation of RNA polymerase III controls tissue and organismal growth in Drosophila. EMBO J 31: 1916–1930. doi: 10.1038/emboj.2012.33 22367393 10.1038/emboj.2012.33 PMC3343326

[40] Rideout EJ, Marshall L, Grewal SS (2012) Drosophila RNA polymerase III repressor Maf1 controls body size and developmental timing by modulating tRNAiMet synthesis and systemic insulin signalling. Proc Natl Acad Sci U S A 109: 1139–1144. doi: 10.1073/pnas.1113311109 22228302 10.1073/pnas.1113311109 PMC3268294

[41] Zhong S, Zhang C, Johnson DL (2004) Epidermal growth factor enhances cellular TATA binding protein levels and induces RNA polymerase I- and III-dependent gene activity. Mol Cell Biol 24: 5119–5129. doi: 10.1128/MCB.24.12.5119-5129.2004 15169879 10.1128/MCB.24.12.5119-5129.2004 PMC419868

[42] Geiduschek EP, Kassavetis GA (2001) The RNA polymerase III transcription apparatus. J Mol Biol 310: 1–26. doi: 10.1006/jmbi.2001.4732 11419933 10.1006/jmbi.2001.4732

[43] Cordero JB, Stefanatos RK, Myant K, Vidal M, Sansom OJ (2012) Non-autonomous crosstalk between the Jak/Stat and Egfr pathways mediates Apc1-driven intestinal stem cell hyperplasia in the Drosophila adult midgut. Development 139: 4524–4535. doi: 10.1242/dev.078261 23172913 10.1242/dev.078261

[44] Steiger D, Furrer M, Schwinkendorf D, Gallant P (2008) Max-independent functions of Myc in Drosophila melanogaster. Nat Genet 40: 1084–1091. doi: 10.1038/ng.178 19165923 10.1038/ng.178

[45] Gomez-Roman N, Grandori C, Eisenman RN, White RJ (2003) Direct activation of RNA polymerase III transcription by c-Myc. Nature 421: 290–294. doi: 10.1038/nature01327 12529648 10.1038/nature01327

[46] Sears R, Leone G, DeGregori J, Nevins JR (1999) Ras enhances Myc protein stability. Mol Cell 3: 169–179. 10078200 10.1016/s1097-2765(00)80308-1

[47] Soucek L, Whitfield JR, Sodir NM, Masso-Valles D, Serrano E, et al (2013) Inhibition of Myc family proteins eradicates KRas-driven lung cancer in mice. Genes Dev 27: 504–513. doi: 10.1101/gad.205542.112 23475959 10.1101/gad.205542.112 PMC3605464

[48] Kantidakis T, Ramsbottom BA, Birch JL, Dowding SN, White RJ (2010) mTOR associates with TFIIIC, is found at tRNA and 5S rRNA genes, and targets their repressor Maf1. Proc Natl Acad Sci U S A 107: 11823–11828. doi: 10.1073/pnas.1005188107 20543138 10.1073/pnas.1005188107 PMC2900655

[49] Michels AA, Robitaille AM, Buczynski-Ruchonnet D, Hodroj W, Reina JH, et al (2010) mTORC1 directly phosphorylates and regulates human MAF1. Mol Cell Biol 30: 3749–3757. doi: 10.1128/MCB.00319-10 20516213 10.1128/MCB.00319-10 PMC2916396

[50] Shor B, Wu J, Shakey Q, Toral-Barza L, Shi C, et al (2010) Requirement of the mTOR kinase for the regulation of Maf1 phosphorylation and control of RNA polymerase III-dependent transcription in cancer cells. J Biol Chem 285: 15380–15392. doi: 10.1074/jbc.M109.071639 20233713 10.1074/jbc.M109.071639 PMC2865278

[51] Wei Y, Tsang CK, Zheng XF (2009) Mechanisms of regulation of RNA polymerase III-dependent transcription by TORC1. EMBO J 28: 2220–2230. doi: 10.1038/emboj.2009.179 19574957 10.1038/emboj.2009.179 PMC2726700

[52] Moir RD, Lee J, Haeusler RA, Desai N, Engelke DR, et al (2006) Protein kinase A regulates RNA polymerase III transcription through the nuclear localization of Maf1. Proc Natl Acad Sci U S A 103: 15044–15049. doi: 10.1073/pnas.0607129103 17005718 10.1073/pnas.0607129103 PMC1622776

[53] Huber A, Bodenmiller B, Uotila A, Stahl M, Wanka S, et al (2009) Characterization of the rapamycin-sensitive phosphoproteome reveals that Sch9 is a central coordinator of protein synthesis. Genes Dev 23: 1929–1943. doi: 10.1101/gad.532109 19684113 10.1101/gad.532109 PMC2725941

[54] Oficjalska-Pham D, Harismendy O, Smagowicz WJ, Gonzalez de Peredo A, Boguta M, et al (2006) General repression of RNA polymerase III transcription is triggered by protein phosphatase type 2A-mediated dephosphorylation of Maf1. Mol Cell 22: 623–632. doi: 10.1016/j.molcel.2006.04.008 16762835 10.1016/j.molcel.2006.04.008

[55] Roberts DN, Wilson B, Huff JT, Stewart AJ, Cairns BR (2006) Dephosphorylation and genome-wide association of Maf1 with Pol III-transcribed genes during repression. Mol Cell 22: 633–644. doi: 10.1016/j.molcel.2006.04.009 16762836 10.1016/j.molcel.2006.04.009 PMC2788557

[56] Oler AJ, Cairns BR (2012) PP4 dephosphorylates Maf1 to couple multiple stress conditions to RNA polymerase III repression. EMBO J 31: 1440–1452. doi: 10.1038/emboj.2011.501 22333918 10.1038/emboj.2011.501 PMC3321174

[57] Felton-Edkins ZA, Kenneth NS, Brown TR, Daly NL, Gomez-Roman N, et al (2003) Direct regulation of RNA polymerase III transcription by RB, p53 and c-Myc. Cell Cycle 2: 181–184. 12734418

[58] Polymenis M, Schmidt EV (1997) Coupling of cell division to cell growth by translational control of the G1 cyclin CLN3 in yeast. Genes Dev 11: 2522–2531. 9334317 10.1101/gad.11.19.2522 PMC316559

[59] Dowling RJ, Topisirovic I, Alain T, Bidinosti M, Fonseca BD, et al (2010) mTORC1-mediated cell proliferation, but not cell growth, controlled by the 4E-BPs. Science 328: 1172–1176. doi: 10.1126/science.1187532 20508131 10.1126/science.1187532 PMC2893390

[60] Hsieh AC, Liu Y, Edlind MP, Ingolia NT, Janes MR, et al (2012) The translational landscape of mTOR signalling steers cancer initiation and metastasis. Nature 485: 55–61. doi: 10.1038/nature10912 22367541 10.1038/nature10912 PMC3663483

[61] Morita M, Gravel SP, Chenard V, Sikstrom K, Zheng L, et al (2013) mTORC1 controls mitochondrial activity and biogenesis through 4E-BP-dependent translational regulation. Cell Metab 18: 698–711. doi: 10.1016/j.cmet.2013.10.001 24206664 10.1016/j.cmet.2013.10.001

[62] Thoreen CC, Chantranupong L, Keys HR, Wang T, Gray NS, et al (2012) A unifying model for mTORC1-mediated regulation of mRNA translation. Nature 485: 109–113. doi: 10.1038/nature11083 22552098 10.1038/nature11083 PMC3347774

[63] Grewal SS (2015) Why should cancer biologists care about tRNAs? tRNA synthesis, mRNA translation and the control of growth. Biochim Biophys Acta 1849: 898–907. doi: 10.1016/j.bbagrm.2014.12.005 25497380 10.1016/j.bbagrm.2014.12.005

[64] Zhou Y, Goodenbour JM, Godley LA, Wickrema A, Pan T (2009) High levels of tRNA abundance and alteration of tRNA charging by bortezomib in multiple myeloma. Biochem Biophys Res Commun 385: 160–164. doi: 10.1016/j.bbrc.2009.05.031 19450555 10.1016/j.bbrc.2009.05.031 PMC2774282

[65] Pavon-Eternod M, Gomes S, Geslain R, Dai Q, Rosner MR, et al (2009) tRNA over-expression in breast cancer and functional consequences. Nucleic Acids Res 37: 7268–7280. doi: 10.1093/nar/gkp787 19783824 10.1093/nar/gkp787 PMC2790902

[66] Gingold H, Tehler D, Christoffersen NR, Nielsen MM, Asmar F, et al (2014) A dual program for translation regulation in cellular proliferation and differentiation. Cell 158: 1281–1292. doi: 10.1016/j.cell.2014.08.011 25215487 10.1016/j.cell.2014.08.011

[67] Goodarzi H, Nguyen HC, Zhang S, Dill BD, Molina H, et al (2016) Modulated Expression of Specific tRNAs Drives Gene Expression and Cancer Progression. Cell 165: 1416–1427. doi: 10.1016/j.cell.2016.05.046 27259150 10.1016/j.cell.2016.05.046 PMC4915377

[68] Clarke CJ, Berg TJ, Birch J, Ennis D, Mitchell L, et al (2016) The Initiator Methionine tRNA Drives Secretion of Type II Collagen from Stromal Fibroblasts to Promote Tumor Growth and Angiogenesis. Curr Biol 26: 755–765. doi: 10.1016/j.cub.2016.01.045 26948875 10.1016/j.cub.2016.01.045 PMC4819511

[69] Birch J, Clarke CJ, Campbell AD, Campbell K, Mitchell L, et al (2016) The initiator methionine tRNA drives cell migration and invasion leading to increased metastatic potential in melanoma. Biol Open 5: 1371–1379. doi: 10.1242/bio.019075 27543055 10.1242/bio.019075 PMC5087684

[70] Pavon-Eternod M, Gomes S, Rosner MR, Pan T (2013) Overexpression of initiator methionine tRNA leads to global reprogramming of tRNA expression and increased proliferation in human epithelial cells. RNA 19: 461–466. doi: 10.1261/rna.037507.112 23431330 10.1261/rna.037507.112 PMC3677255

[71] Rojas-Benitez D, Thiaville PC, de Crecy-Lagard V, Glavic A (2015) The Levels of a Universally Conserved tRNA Modification Regulate Cell Growth. J Biol Chem 290: 18699–18707. doi: 10.1074/jbc.M115.665406 26063805 10.1074/jbc.M115.665406 PMC4513126

[72] Grewal SS, Li L, Orian A, Eisenman RN, Edgar BA (2005) Myc-dependent regulation of ribosomal RNA synthesis during Drosophila development. Nat Cell Biol 7: 295–302. doi: 10.1038/ncb1223 15723055 10.1038/ncb1223

[73] Ashton-Beaucage D, Udell CM, Gendron P, Sahmi M, Lefrancois M, et al (2014) A functional screen reveals an extensive layer of transcriptional and splicing control underlying RAS/MAPK signalling in Drosophila. PLoS Biol 12: e1001809 doi: 10.1371/journal.pbio.1001809 24643257 10.1371/journal.pbio.1001809 PMC3958334

[74] Bellosta P, Hulf T, Balla Diop S, Usseglio F, Pradel J, et al (2005) Myc interacts genetically with Tip48/Reptin and Tip49/Pontin to control growth and proliferation during Drosophila development. Proc Natl Acad Sci U S A 102: 11799–11804. doi: 10.1073/pnas.0408945102 16087886 10.1073/pnas.0408945102 PMC1187951

