# Supplementary figures and images for: Ras/ERK-signalling promotes tRNA synthesis and growth via the RNA polymerase III repressor Maf1 in Drosophila
Source: PLoS Genet. 2018 Feb 5;14(2):e1007202. doi: 10.1371/journal.pgen.1007202 (PMC5814106; doi:10.1371/journal.pgen.1007202)

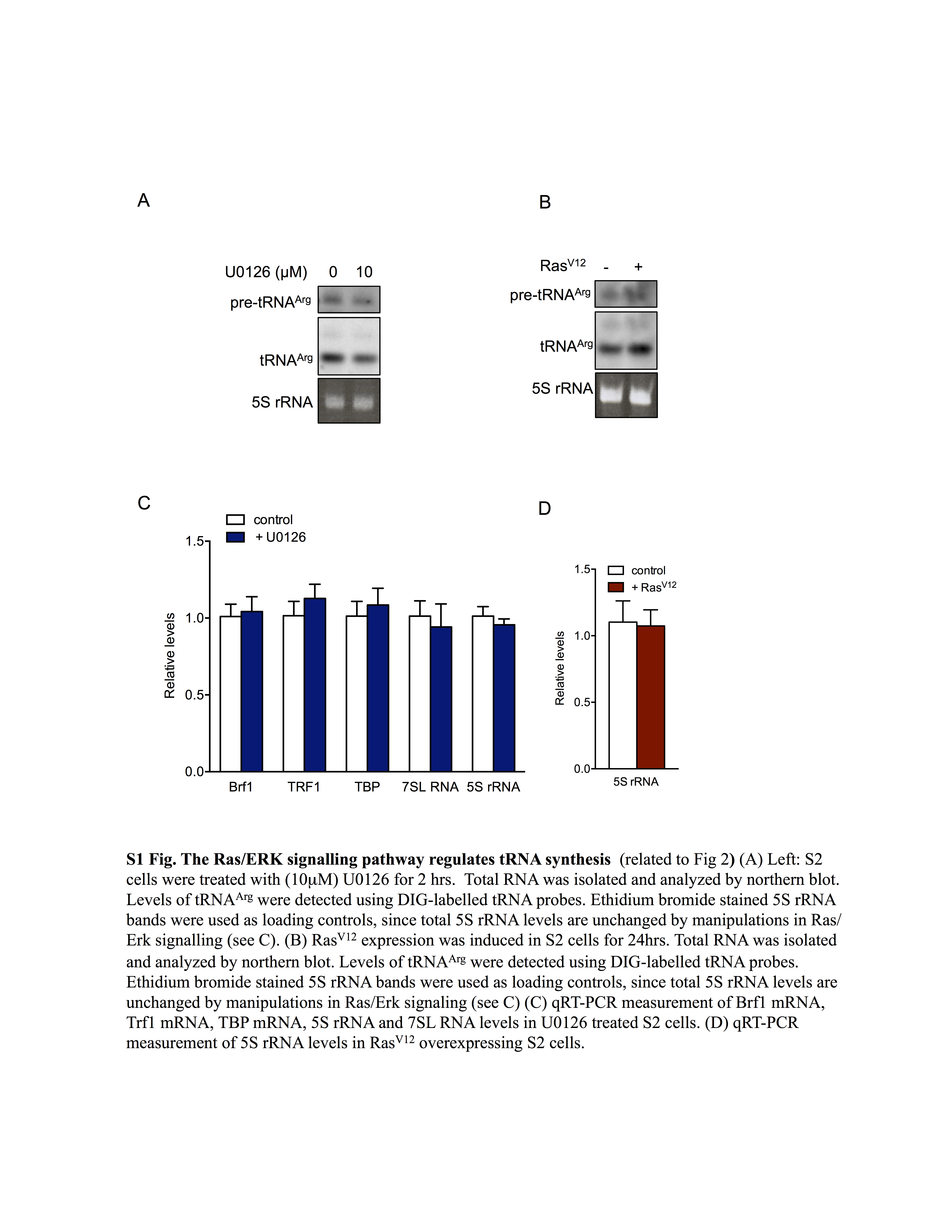

Supplement: S1 Fig — (A) Left: S2 cells were treated with (10μM) U0126 for 2 hrs. Total RNA was isolated and analyzed by Northern blot. Levels of tRNAArg were detected using DIG-labelled tRNA probes. Ethidium bromide stained 5S rRNA bands were used as loading controls, since total 5S rRNA levels are unchanged by manipulations in Ras/Erk signalling (see C). (B) RasV12 expression was induced in S2 cells for 24hrs. Total RNA was isolated and analyzed by northern blot. Levels of tRNAArg were detected using DIG-labelled tRNA probes. Ethidium bromide stained 5S rRNA bands were used as loading controls, since total 5S rRNA levels are unchanged by manipulations in Ras/Erk signalling (see C) (C) qRT-PCR measurement of Brf1 mRNA, Trf1 mRNA, TBP mRNA, 5S rRNA and 7SL RNA levels in U0126 treated S2 cells. (D) qRT-PCR measurement of 5S rRNA levels in RasV12 overexpressing S2 cells. (TIFF) [file pgen.1007202.s001.tiff]

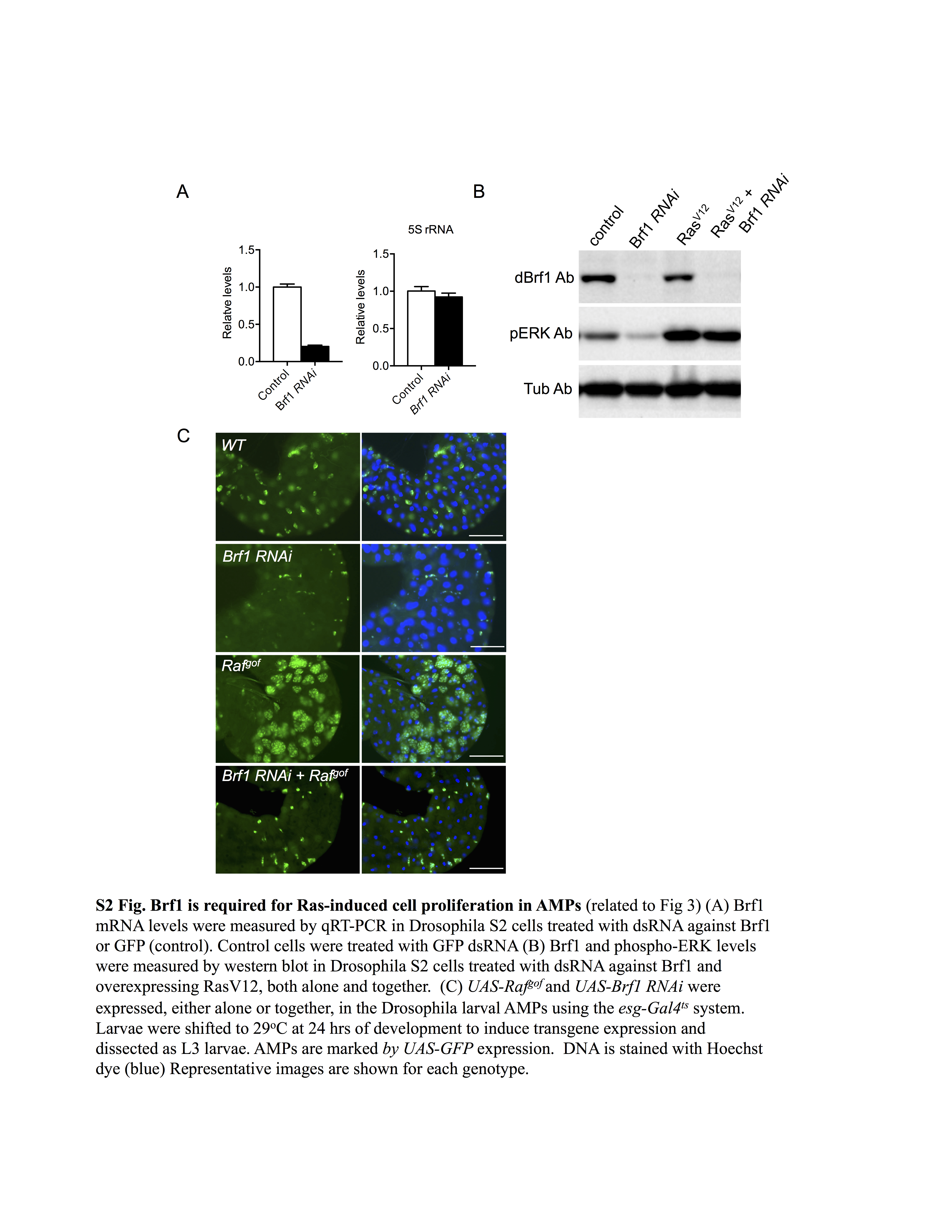

Supplement: S2 Fig — (A) Brf1 mRNA levels were measured by qRT-PCR in Drosophila S2 cells treated with dsRNA against Brf1 or GFP (control). Control cells were treated with GFP dsRNA (B) Brf1, phospho-ERK levels and alpha-tubulin protein levels were measured by western blot in Drosophila S2 cells treated with dsRNA against Brf1 and overexpressing RasV12, both alone and together. (C) UAS-Rafgof and UAS-Brf1 RNAi were expressed, either alone or together, in the Drosophila larval AMPs using the esg-Gal4ts system. Larvae were shifted to 29°C at 24 hrs of development to induce transgene expression and dissected as L3 larvae. AMPs are marked by UAS-GFP expression. DNA is stained with Hoechst dye (blue) Representative images are shown for each genotype. (TIFF) [file pgen.1007202.s002.tiff]

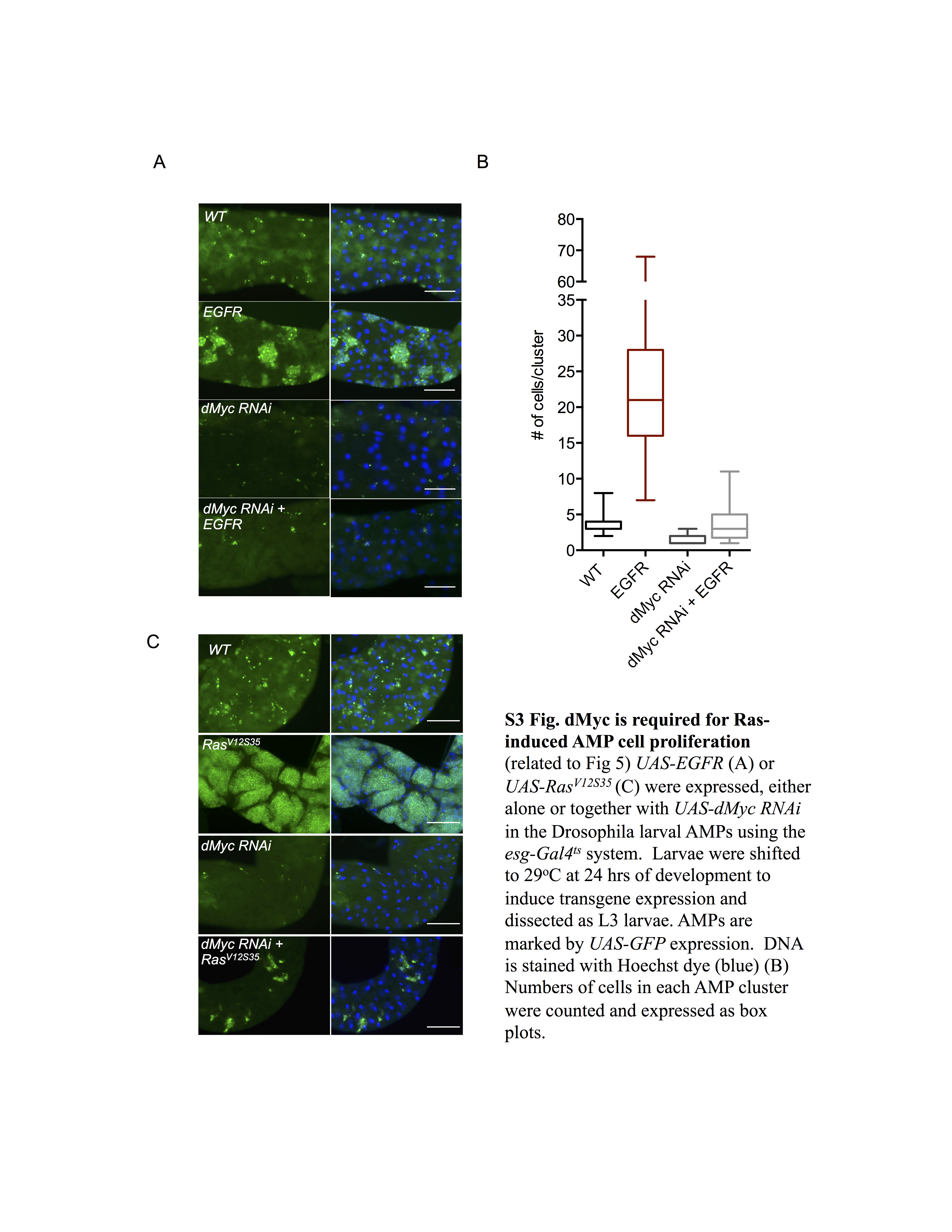

Supplement: S3 Fig — UAS-EGFR (A) or UAS-RasV12S35 (C) were expressed, either alone or together with UAS-dMyc RNAi in the Drosophila larval AMPs using the esg-Gal4ts system. Larvae were shifted to 29°C at 24 hrs of development to induce transgene expression and dissected as L3 larvae. AMPs are marked by UAS-GFP expression. DNA is stained with Hoechst dye (blue) (B) (related to experiment in A) Numbers of cells in each AMP cluster were counted and expressed as box plots. (TIFF) [file pgen.1007202.s003.tiff]

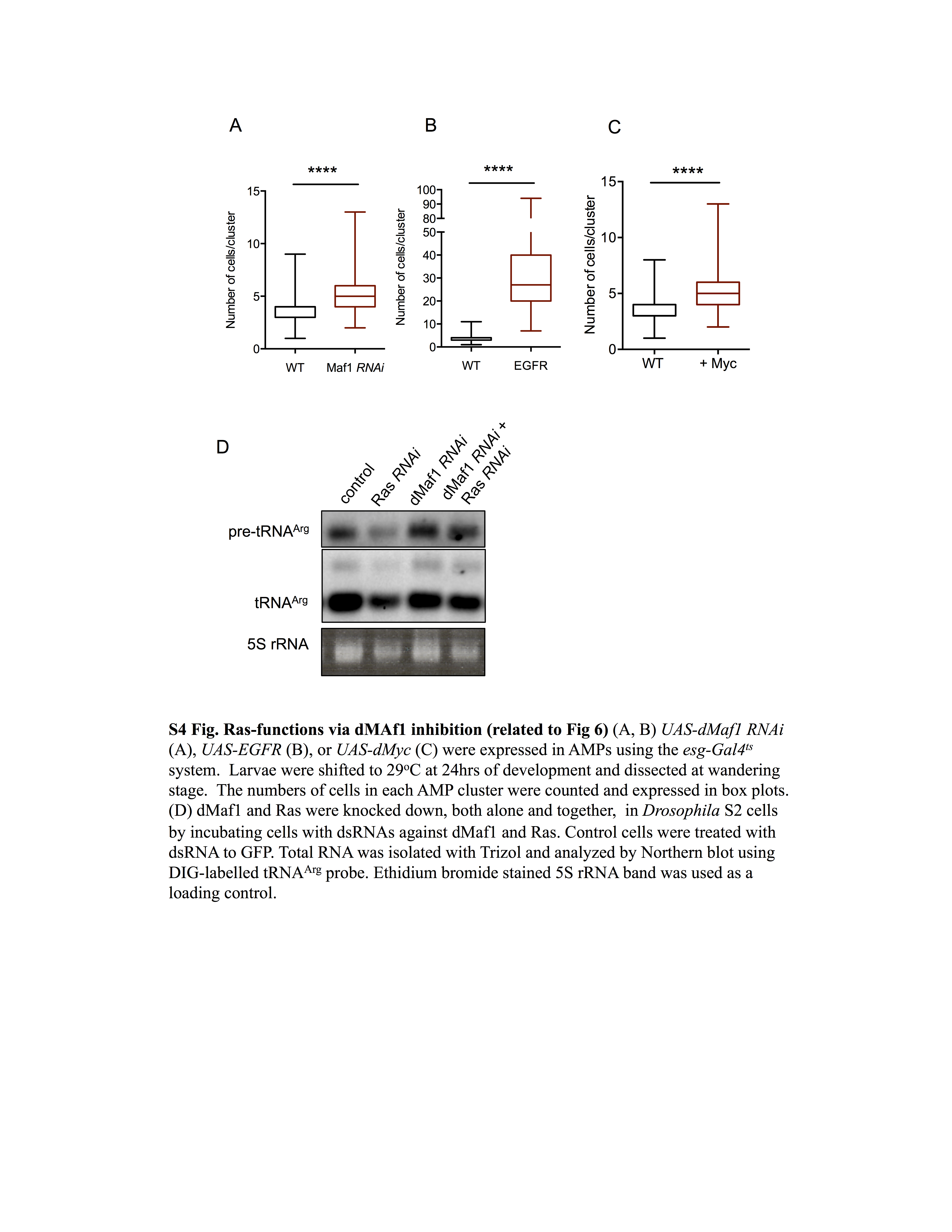

Supplement: S4 Fig — (A, B) UAS-dMaf1 RNAi (A), UAS-EGFR (B), or UAS-dMyc (C) were expressed in AMPs using the esg-Gal4ts system. Larvae were shifted to 29°C at 24hrs of development and dissected at wandering stage. The numbers of cells in each AMP cluster were counted and expressed in box plots. (D) dMaf1 and Ras were knocked down, both alone and together, in Drosophila S2 cells by incubating cells with dsRNAs against dMaf1 and Ras. Control cells were treated with dsRNA to GFP. Total RNA was isolated with Trizol and analyzed by Northern blot using DIG-labelled tRNAArg probe. Ethidium bromide stained 5S rRNA band was used as a loading control. (TIFF) [file pgen.1007202.s004.tiff]

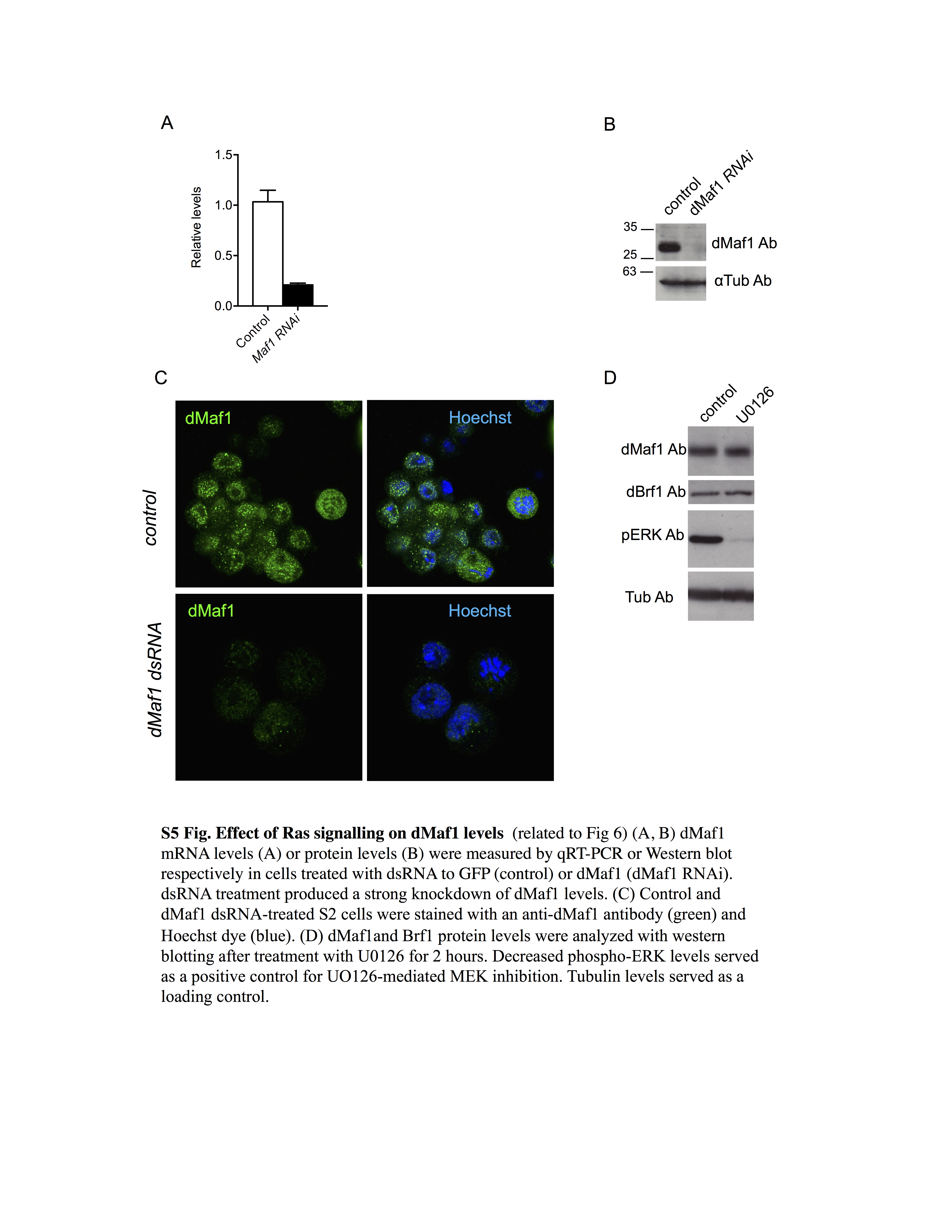

Supplement: S5 Fig — (A, B) dMaf1 mRNA levels (A) or protein levels (B) were measured by qRT-PCR or Western blot respectively in cells treated with dsRNA to GFP (control) or dMaf1 (dMaf1 RNAi). dsRNA treatment produced a strong knockdown of dMaf1 levels. (C) Control and dMaf1 dsRNA-treated S2 cells were stained with an anti-dMaf1 antibody (green) and Hoechst dye (blue). (D) dMaf1and Brf1 protein levels were analyzed with western blotting after treatment with U0126 for 2 hours. Decreased phospho-ERK levels served as a positive control for UO126-mediated MEK inhibition. Tubulin levels served as a loading control. (TIFF) [file pgen.1007202.s005.tiff]

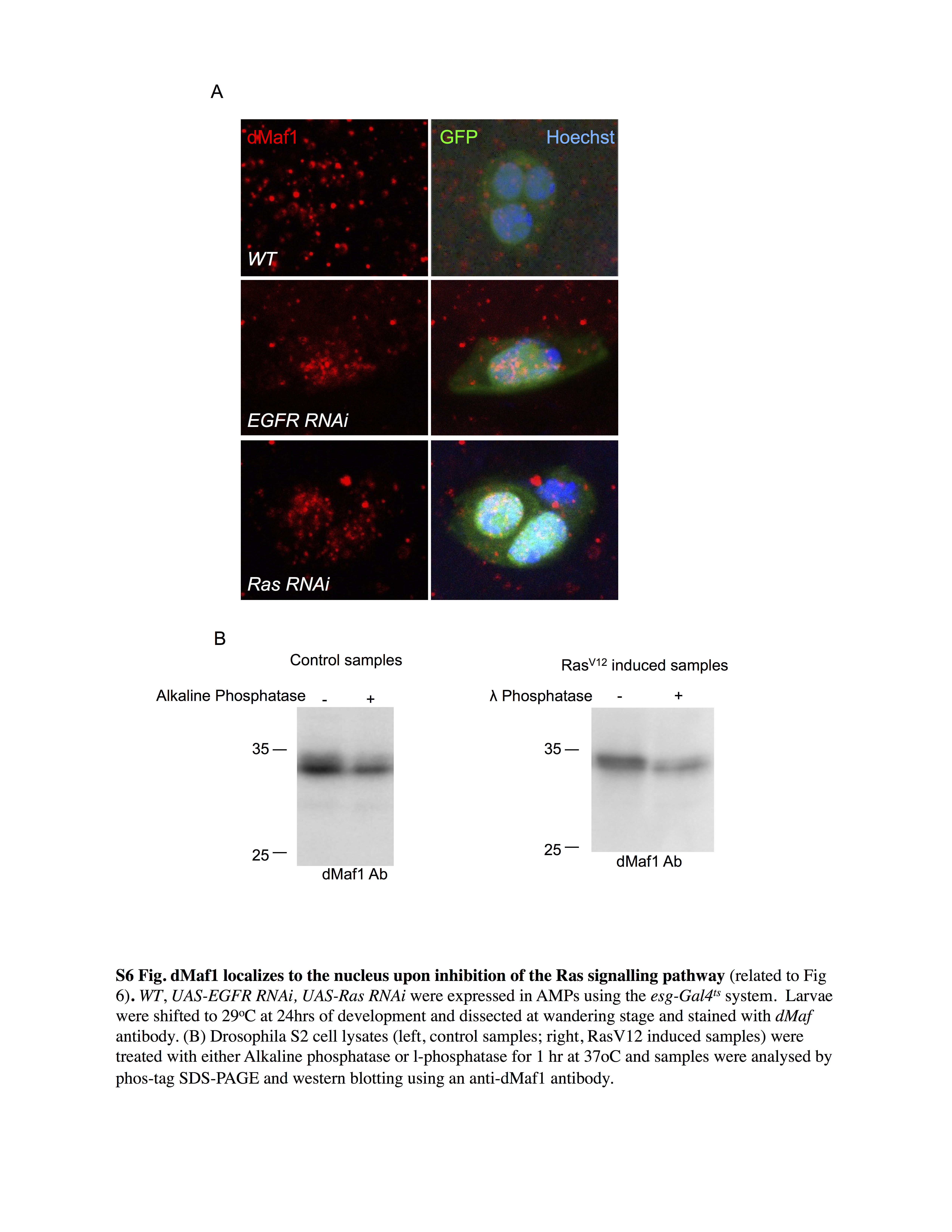

Supplement: S6 Fig — WT, UAS-EGFR RNAi, UAS-Ras RNAi were expressed in AMPs using the esg-Gal4ts system. Larvae were shifted to 29°C at 24hrs of development and dissected at wandering stage and stained with dMaf1 antibody. (B) Drosophila S2 cell lysates (left, control samples; right, RasV12 induced samples) were treated with either Alkaline phosphatase or λ-phosphatase for 1 hr at 37°C and samples were analysed by phos-tag SDS-PAGE and western blotting using an anti-dMaf1 antibody. (TIFF) [file pgen.1007202.s006.tiff]

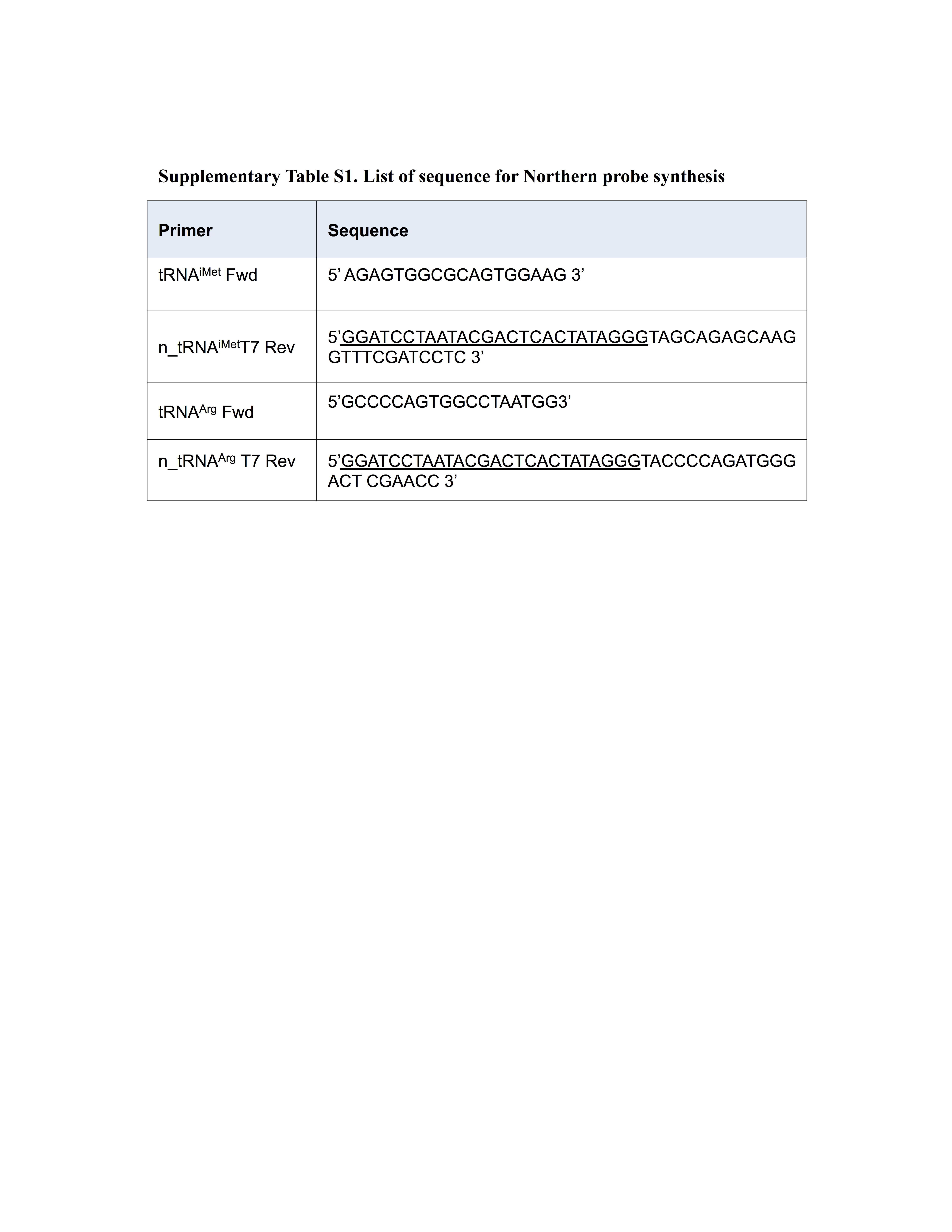

Supplement: S1 Table — (TIFF) [file pgen.1007202.s007.tiff]

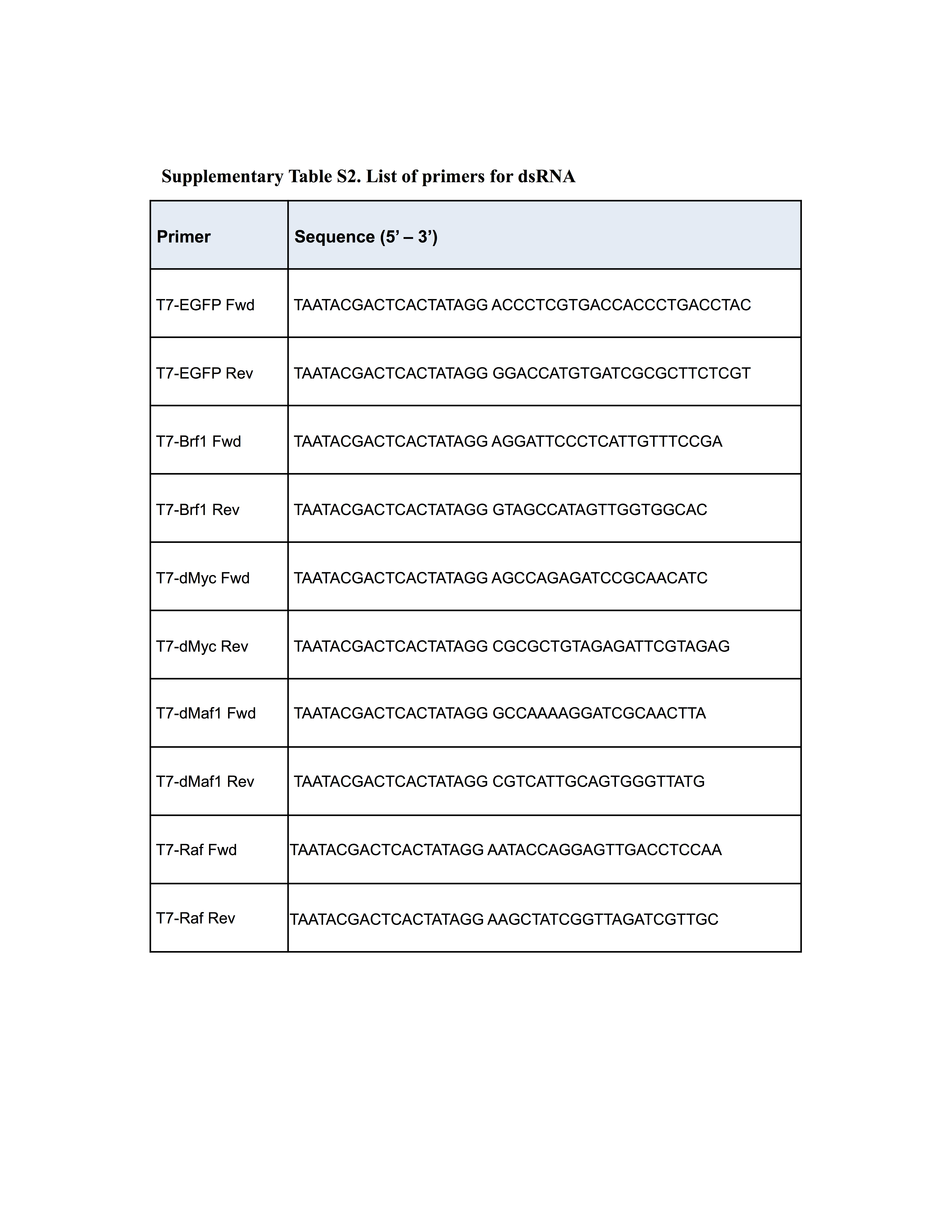

Supplement: S2 Table — (TIFF) [file pgen.1007202.s008.tiff]

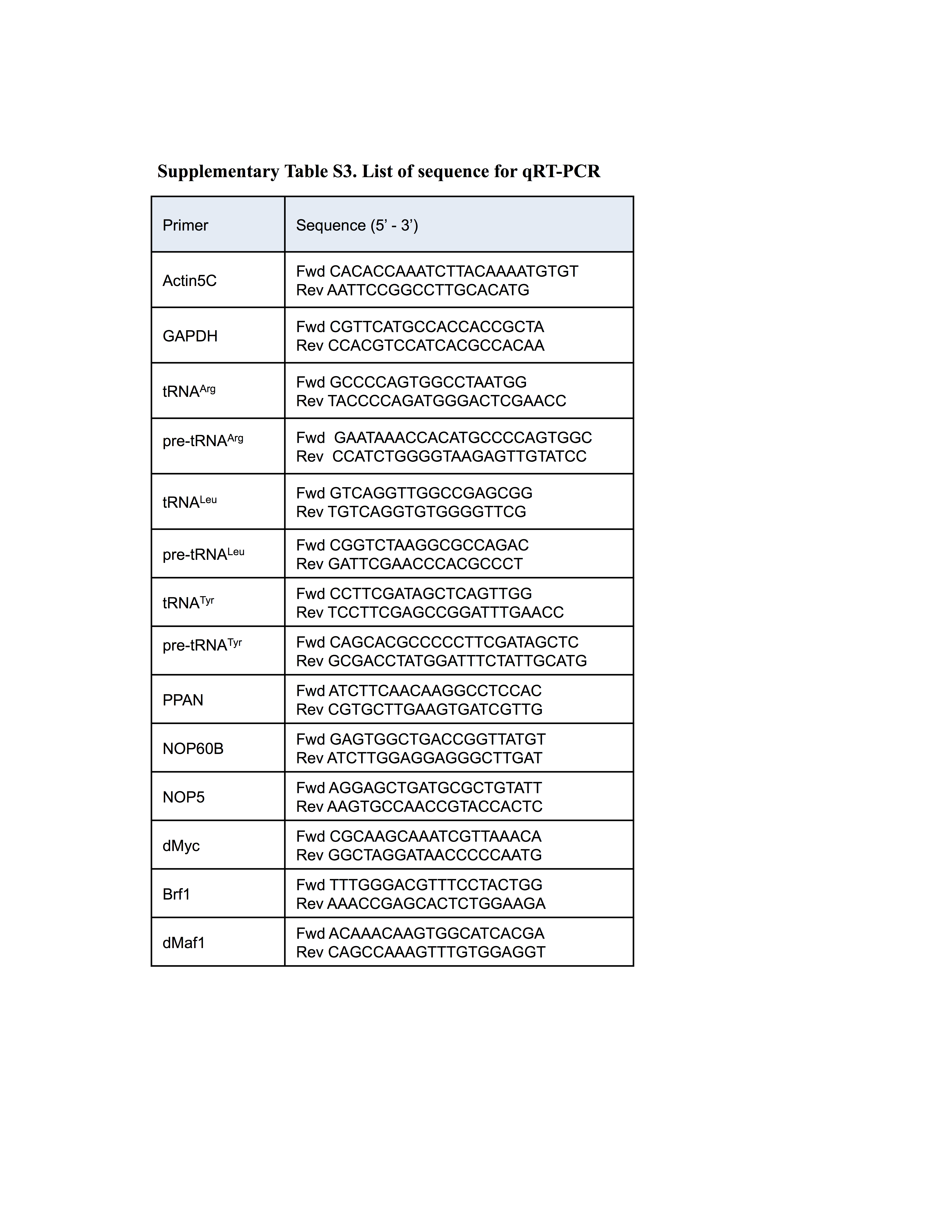

Supplement: S3 Table — (TIFF) [file pgen.1007202.s009.tiff]
